# Enhancing CAR-T Cell Metabolic Fitness and Memory Phenotype for Improved Efficacy against Hepatocellular Carcinoma

**DOI:** 10.7150/ijbs.110406

**Published:** 2025-06-20

**Authors:** Jinqi You, Xinyi Yang, Jingjing Zhao, Hao Chen, Yan Tang, Dijun Ouyang, Yuanyuan Liu, Yan Wang, Songzuo Xie, Yuanyuan Chen, Jinghao Liao, Tong Xiang, Jianchuan Xia, Chaopin Yang, Desheng Weng

**Affiliations:** 1 State Key Laboratory of Oncology in South China, Guangdong Provincial Clinical Research Center for Cancer, Collaborative Innovation Center for Cancer Medicine, Sun Yat-sen University Cancer Center, Guangzhou, Guangdong, 510060, P. R. China.; 2 Department of Biotherapy, Sun Yat-sen University Cancer Center, Guangzhou, Guangdong, 510060, P. R. China.

**Keywords:** PD-1 scFv-secreting and CD133-specific CAR-T, metformin, metabolic fitness, mitochondria, memory, STAT3/STAT5

## Abstract

The persistence of chimeric antigen receptor (CAR) T cells in the tumor microenvironment limits their antitumor effects against solid tumors. Many studies have reported that the *in vitro* phenotype and metabolism of CAR-T cells correlates with their *in vivo* antitumor activity. Herein, we constructed PD-1 scFv-secreting and CD133-specific CAR-T (referred to as CAR-T) cells based on our previous work. We found that suitable concentration metformin-treated CAR-T (mCAR-T) cells exhibited an increased memory phenotype and demonstrated stronger and faster antitumor abilities with a reduced exhaustion phenotype. Using RNA sequencing, transmission electron microscope, and metabolic analysis, we discovered enhanced mitochondrial biogenesis and metabolism in CAR-T cells treated with 10 μM metformin, is associated with increased peroxisome proliferator-activated receptor gamma coactivator-1alpha (PGC-1α) expression, promotion of signal transducer and activator of transcription (STAT)3 and inhibition of STAT5 phosphorylation. This resulted in enhanced antitumor effects of mCAR-T cells in both subcutaneous and orthotopic xenograft models. Importantly, in some relapsed hepatocellular carcinoma (HCC) patients, high CD133 expression was observed in their paired primary or metastatic tumor sections. Our study revealed that enhancing metabolic fitness and central memory by *in vitro* metformin treatment is an effective strategy to improve the efficacy of CAR-T cell therapy, potentially benefiting some relapsed HCC patients

## Introduction

Chimeric antigen receptor (CAR) T-cell therapy has shown remarkable efficacy in treating hematopoietic tumors^1^. However, relapse in patients has been reported in clinical trial^2^, ^3^. Additionally, CAR-T cells have demonstrated limited effectiveness against solid tumors including liver cancer^4^-^6^. A hostile tumor microenvironment (TME) is a major obstacle to CAR-T cell survival in solid tumors. To address this, various strategies have been employed to enhance the capabilities of infiltrating T cells^7^, ^8^, such as secreting checkpoint antibodies like PD-1^9^, ^10^ and CD47^11^, ^12^. While these approaches have shown improvements in antitumor abilities^10^, T cells still face the metabolic challenges when competing with tumor cells for nutrients in the TME. Nutrient deprivation (low glucose, decreased amino acids, and reduced oxidative phosphorylation), hypoxia, and accumulation of lactic acid and relative oxygen species (ROS) pose significant hurdles^13^-^15^.

Mitochondria-related metabolism is crucial for CD8^+^ T cell fate decisions and antitumor immunity^16^. T cells within the TME undergo metabolic or functional exhaustion, which is characterized by dysfunctional mitochondria, decreased total mitochondrial mass, fragmented morphology, increased number of depolarized mitochondria, and elevated ROS levels. Modulating mitochondrial metabolism represent an attractive strategy for improving cancer immunotherapy, such as overexpressing OPA1, PGC-1α and MFN2 to enhance T cell mitochondrial biogenesis and mitofusion^17^-^22^. However, CAR-T cell production currently relies on viral transfection and electroporation. The limited cargo capacity is a significant obstacle in manufacturing CAR-T cells with large protein expression, such as PGC-1α. Without complex preparation processes, the pharmacological induction of mitochondrial biogenesis in T cells may be another potential therapeutic target for cancer immunotherapy. For example, using Bezafibrate, a PGC-1α agonist, has demonstrated enhanced antitumor immunity by upregulating mitochondrial oxidative phosphorylation (OXPHOS) and inhibiting apoptosis in a mouse tumor model (MC38) with PD-1 blockade^23^. Treatment with the phosphatidylinositol-3-kinase δ/γ inhibitor duvelisib increases MFN2 expression in CD8^+^ CAR-T cells^24^. Therefore, the exploration of new drugs that enhance CAR-T cell activity is promising.

Metformin, a first-line therapy for type 2 diabetes, is recognized as a metabolic regulator and has exhibited antitumor effects in numerous clinical trials. The combined antitumor effects of metformin and PD-1 antibodies have demonstrated therapeutic benefits in humans and mice^25^-^27^. Patients with diabetes also exhibit reduced tumor incidence in various cancer clinical trials^28^. Metformin modulates the TME by diminishing the intratumoral accumulation of myeloid-derived suppressor cells, downregulating PD-L1 expression, and enhancing autophagy by reducing OXPHOS via mitochondrial complex 1 targeting^29^-^35^. However, the mechanisms underlying these antitumor effects remain elusive.

Emerging studies have shown that *in vitro* T cells with memory-like characteristics exhibit superior *in vivo* antitumor effect^36^-^41^. In this study, we found that treating CAR-T cells with Metformin *in vitro* enhanced mitochondrial biogenesis and maintained their memory phenotype, ultimately improving their *in vivo* antitumor abilities.

## Materials and Methods

### Cell lines

The human hepatocellular carcinoma (HCC) cell line Hep3B, SK-HEP-1 and the human embryonic kidney cell line HEK293T, were obtained from ATCC and identified using short tandem repeat (STR) profiling. All cell lines were cultured in Dulbecco's modified Eagle's medium (DMEM; Gibco) supplemented with 10% fetal bovine serum (FBS; Gibco) at 37 °C in a humid atmosphere containing 5% CO2.

### Animals

NOD-Prkdc^em26Cd52^il2rg^em26Cd22^/Nju (NCG) mice aged 4-6 weeks were purchased from Gempharmatech Co., Ltd, and subsequently housed and subjected to experiments within an SPF-grade animal laboratory situated at the Animal Experiment Center, North Campus, Sun Yat-sen University.

### Patient sample collection

Paraffin-embedded paired primary and metastatic tumor tissues of 9 HCC patients who had received conventional therapies and relapsed were obtained from Sun Yat-sen University Cancer Center (Guangzhou, P.R. China).

### Lentivirus packaging

The PD-1 scFv-secreting and CD133-specific CAR (PD1 scFv and CD133 CAR) contains a CD133 scFv sequence, CD8 hinge domain, CD28 transmembrane and intra domain, P2A ribosomal skip sequence, and PD-1 scFv with an HA-tag. The CAR genes were cloned into a lentiviral vector. The lentiviruses were produced as previously described^42^, ^43^. Briefly, HEK293T cells were co-transfected with the CAR-expressing lentiviral plasmid, packaging plasmid psPAX2 (Addgene #12260), and envelope plasmid pMD2.G (Addgene #12259) using Lipofectamine^TM^ 2000 Reagent (Invitrogen, Carlsbad, CA,USA). After 52 h, culture supernatants were harvested and concentrated for gene transduction. Lentiviral vectors encoding CD133 (puromycin resistance), luciferase (blasticidin resistance), and PD-1 were generated using the same procedure.

### CAR-T cell construction and expansion *in vitro*

Peripheral blood mononuclear cells were obtained from the blood of healthy donors using MD Pacific Cell Separation Media (MD Pacific, Tianjin, China). Next, T cells were isolated using Pan T Cell Isolation Kit (Miltenyi, Bergisch Gladbach, Germany) and stimulated with ImmunoCult™ Human CD3/CD28 T Cell Activator (Stemcell, USA) for 48 h. Lentiviral vector encoding PD1 scFv and CD133 CAR was used to infect activated T cells to construct CAR-T cells. Un-transduced T cells and transduced CAR-T cells were cultured in X-VIVO-15 medium (Lonza) supplemented with 300 U/ml recombinant human IL-2 (Four Rings Biopharmaceutical, Beijing, China). For mCAR-T and mNT cells, different concentrations of metformin (10, 100, or 1000 μM) (Selleck) was added into the medium three times a week. T cells were cultured and expanded *in vitro* for analysis and treatment of tumor-bearing mice.

### Lentiviral transduction to express CD133, luciferase and PD-1

For lentiviral transduction of tumor cells, lentivirus encoding CD133 or luciferase was added to SK-HEP-1 or CD133-SK-HEP-1 cells in the presence of 8 μg/ml polybrene (MCE, Cat# HY-112735) to enhance transduction efficiency. The positive tumor cells were selected using puromycin or blasticidin after 48 h. To generate PD-1-overexpressing T cells, primary T cells from healthy donors were transduced with lentivirus encoding PD-1, and the positive cells were enriched using fluorescence-activated cell sorting (FACS).

### CRISPR/Cas9-mediated knockout of CD133

To generate CD133-knockout (CD133-KO) Hep3B cells, wild-type Hep3B cells were transfected with the CD133 CRISPR/Cas9 KO Plasmid (Santa Cruz Biotechnology, Cat# sc-418263) using Lipofectamine™ 3000 Transfection Reagent (Thermo Fisher Scientific) according to the manufacturer's instructions. Briefly, 2×10^5^ Hep3B cells per well were seeded into a 6-well plate. After 24 h, 1 µg of CD133-KO plasmid was diluted in 125 µl Opti-MEM medium, followed by addition of 2 µl P3000 reagent. Separately, 3.7 µl Lipofectamine 3000 was diluted in 125 µl Opti-MEM. The solutions were mixed and incubated at room temperature for 10-15 min, then added to the seeded Hep3B cells. After 24 h of transfection, the medium was replaced with fresh complete medium. FACS was performed to enrich the CD133-negative cells.

### Flow cytometry

To evaluate the binding activity of the PD-1 blocking scFv, the culture medium from CAR-T cells was collected and concentrated, and then different volumes (5 µl, 10 µl, 20 µl, and 50 µl) of the concentrated medium were incubated with PD-1-overexpressing T and NT cells at 4 °C for 40 min. PE-conjugated anti-tag antibody was used to detect the tagged PD-1 scFv bound to T cells.

CAR expression in the transduced T cells was detected using human CD133 protein with a His-tag (Acro biosystem, Cat# CD3-H52H4) and PE anti-His-tag antibody (BioLegend, Cat# 362603). BUV395 anti-human CD3 (BD Biosciences, Cat# 563546), FITC anti-human CD4 (BD Biosciences, Cat# 555346), APC anti-human CD8 (BioLegend, Cat# 344722), PE/Cyanine5 anti-human CD62L (BD Biosciences, Cat# 555545), and BV421 anti-human CD45RO (BD Biosciences, Cat# 562641) antibodies were used to distinguish native T, central memory T, and effector memory T cells. T cell exhaustion was measured through surface staining with APC anti-human CD279 (PD-1; BD Biosciences, Cat# 558694), BV421 anti-human CD223 (LAG-3; BD Biosciences, Cat# 565720), and PE anti-human CD366 (TIM-3; BD Biosciences, Cat# 563422) antibodies.

For intracellular cytokines staining, NT, CAR-T and mCAR-T cells were stimulated with leukocyte activation cocktail with BD GolgiPlug^TM^ (BD Biosciences, Cat# 550583) and monensin (BioLegend, Cat# 420701) for 5 h, and a Fixation/Permeablization Kit (BD Biosciences, Cat# 554714) was then used according to the manufacture's instruction, followed by staining with APC anti-human IFN-γ (BioLegend, Cat# 502512), PE anti-human TNF-α (BioLegend, Cat# 502909), and BV421 anti-human Granzyme B (BD Biosciences, Cat# 563389) antibodies.

Mouse spleens were harvested and transformed into single-cell suspensions. After red blood cell lysis, FITC anti-mouse CD45 (BioLegend, Cat# 103108), APC/Cyanine7 anti-human CD45 (BD Biosciences, Cat# 557833), and PE anti-human CD3 (BD Biosciences, Cat# 555333) antibodies were used to detect human T cells in mouse spleens.

To measure the lipid content and mitochondrial membrane potential in cells, 1 million NT, CAR-T and mCAR-T cells were incubated with BODIPY 493/503 (MCE, Cat# HY-W090090) and JC-1 (MCE, Cat# HY-15534) working solutions for 1 h. Subsequently, flow cytometry was performed to detect fluorescence.

All data were collected using intelligent flow cytometers (CytoFLEX LX, Beckman) and CytExpert (Beckman). FlowJo version 10 was used for analysis.

### Western blot

To validate the secretion of PD-1 scFv, the culture supernatants of NT, CAR-T, and mCAR-T cells used for western blot analysis were collected and concentrated through centrifugation at 3000×g for 15-30 min at 4 °C using a Millipore system (10 kd). For experiments validating the mechanisms underlying metformin-mediated enhancement of CAR-T cell function, 1×10^7^ cells from NT, CAR-T, mCAR-T (10) and mCAR-T (100) groups were harvested. To further evaluate the role of the ERK/CREB/PGC-1α axis, 5×10^6^ cells from CAR-T, mCAR-T (10), and mCAR-T (10) cells treated with 20 μM ERK inhibitor (iERK), SCH772984 (Selleck), were seeded into 6-well plates and harvested after 48 h. The concentrated supernatants and cells from each group were lysed on ice for 30 min in RIPA (Sigma) buffer containing protease and phosphatase inhibitors. The protein concentration was adjusted to be consistent, and SDS-PAGE sample loading buffer (Beyotime, China) was added. Total proteins were separated on a 4-12% gel and then transferred to a PVDF membrane. PD-1 blocking scFv and other target proteins were detected using antibodies to HA-tag (CST, Cat# 2367, 1:1000), ERK1/2 (CST, Cat# 4695, 1:1000), p-ERK1/2 (CST, Cat# 4370, 1:2000), CREB (CST, Cat# 9197, 1:1000), p-CREB (CST, Cat# 9198, 1:1000), PGC-1α (Abcam, Cat# 191838, 1:2000), STAT3 (CST, Cat# 9139, 1:1000), p-STAT3 (CST, Cat# 9145, 1:2000), STAT5 (CST, Cat# 94205, 1:1000), p-STAT5 (CST, Cat# 4322, 1:1000), and GAPDH (Proteintech, Cat# 10494-1-AP, 1:3000). After overnight incubation at 4 °C, the samples were incubated with anti-rabbit IgG HRP-linked antibody (CST, Cat# 7074S, 1:2000) or anti-mouse IgG HRP-linked antibody (CST, Cat# 7076S, 1:2000). Protein expression was detected using ECL chemiluminescent solution (Applygen, China) and a chemiluminescence imaging system (Bio-Rad, ChemiDoc MP).

### Immunohistochemistry

At the end of the animal study, xenograft tumors were removed, fixed in 4% paraformaldehyde (PFA) overnight, embedded in paraffin, and then processed into slices. For IHC staining, human and mouse tumor tissue sections were incubated at 65 °C for 2 h. After deparaffinization in fresh xylene and hydration using 100%, 95%, 80%, and 50% alcohol, followed by distilled water, antigen retrieval was performed in either pH 9.0 EDTA antigen retrieval solution or pH 6.0 citrate buffer solution according to the manufacture's instruction. Endogenous peroxidase was inactivated using 3% H_2_O_2_, followed by blocking with goat serum. The tumor tissues were then incubated with primary antibodies against human CD133 (Invitrogen, Cat# PA5-82184, 1:250), PD-L1 (Abcam, Cat# 228462, 1:100), CD3 (ZSGB-BIO, Cat# ZA-0503, working solution), and Granzyme B (CST, Cat# 46890, 1:400), following incubation with secondary antibodies and chemical staining (Dako, Agilent). We used a digital pathology slide scanner (KFBIO, Ningbo, China) to obtain the whole scans of these sections, and a microscope (Nikon Eclipse Ni-U) was utilized to obtain representative images.

Quantitative scoring of CD3 and Granzyme B was performed as follows: Digital HALO Software (Indica Labs, Albuquerque, NM, USA) was used to identify tumor regions and calculate the tumor region areas and the number of CD3^+^ and Granzyme B^+^ cells in these regions in the images scanned using the KFBIO digital pathology slide scanner, and then we quantified the number of CD3^+^ and Granzyme B^+^ cells per square millimeter within tumor regions.

Semiquantitative scoring of CD133 was performed in accordance with previous work^10^. Briefly, the extent of IHC staining was assessed as 0, ≤ 1% cancer cells stained; 1, 2-25% stained; 2, 26-50% stained; 3, 51-75% stained; and 4, ≥ 75% stained. For staining intensity, the criteria was as follows: 0, no staining; 1, weak staining; 2, moderate staining; and 3, strong staining. The CD133 expression scores were calculated by multiplying the extent scores and the intensity scores. Five random fields per sample were selected and evaluated by two different pathologists.

### ELISA

The supernatants of NT, CAR-T, and mCAR-T cells co-cultured with SK-HEP-1 cells overexpressing CD133 (CD133-SK-HEP-1 cells) at an effector-to-target (E:T) ratio of 1:1 for 48 h were collected, centrifuged at 2000×g for 10 min, and stored at -80 °C. Cytokine-specific precoated ELISA Kits (Dakewe Biotech) were used to detect the secretion of IFN-γ, TNF-α, and IL-2 according to the manufacturer's instructions.

### Cytotoxicity assays

To evaluate the cytotoxic potential of CAR-T cells in killing target cells *in vitro*, NT, CAR-T, and mCAR-T cells were co-cultured with CD133-SK-HEP-1 cells at E:T ratios of 1:1 and 0.1:1, or with wild-type or CD133-KO Hep3B cells at an E:T ratio of 1:1. Antitumor effects were monitored continuously using an xCELLigence RTCA eSight Real-Time Cell Analyzer (Agilent Technologies, Palo Alto, CA, USA), and xCELLigence RTCA Software Pro and RTCA eSight Software were utilized for representative images capture and data analysis. Ten thousand target cells per well were seeded into an E-Plate VIEW 96 (Agilent, 300601030), and effector cells were added according to the different E:T ratios after 24 h, setting to read cell index values every 15 min and capture images every 2 h for real-time monitoring. The cell index represents the change in electrical impedance, which reflects the number of surviving target cells on the biocompatible microelectrode surfaces. The cell index data of each group represents the mean value of the three wells.

### Metabolic analysis

To evaluate the energy metabolism of NT, CAR-T, and mCAR-T cells expanded *in vitro*, Seahorse assay was performed to measure extracellular acidification rate (ECAR) and oxygen consumption rate (OCR). Reagents and products used in the assay were purchased from Agilent Technologies. The probe plate was hydrated, and the equipment and analysis software were preheated the day before the experiment. On the day of the assay, T cells from each group were suspended in Seahorse XF Base Medium supplemented with 10 mM glucose, 1 mM pyruvate, and 2 mM glutamine (no glucose was added to the medium for ECAR); the pH of the medium was adjusted to 7.4. T cells (4×10^5^) per well were seeded in the plate, and ECAR was measured following injections of glucose (10 mM), oligomycin (1 μM), and 2-deoxy-D-glucose (2-DG) (50 mM). Oligomycin (1.5 μM), FCCP (1 μM), rotenone and antimycin A (0.5 μM each) were added for OCR measuring. Data were collected using a Seahorse XF96 Cell Energy Metabolism Analyzer (Agilent) and analyzed using Seahorse Wave Controller Software (Agilent). Both ECAR and OCR measurements were normalized to cell numbers.

### Transmission electron microscope

NT, CAR-T, and mCAR-T (10) cells, totaling 1.5×10^7^ cells each, were fixed using an electron microscope fixing solution containing 2.5% glutaraldehyde at 4 °C for 24 h. After washing in 0.1 M PBS, these cells were treated with 0.1% Millipore-filtered cacodylate-buffered tannic acid, followed by postfixation with 1% buffered osmium and staining with 1% Millipore-filtered uranyl acetate. Subsequently, cells from each group were incubated at 60°C for 48 h and prepared as 60-80 nm slices following dehydration and embedding. Digital images were obtained using a transmission electron microscope (FEI, Tecnai G2 20 S-TWIN). We randomly selected 21 visual fields from each group and utilized ImageJ for quantitative analysis, obtaining and calculating the total T-cell cross-sectional area, mitochondrial number and area per cell, and mitochondrial cross-sectional area relative to the total T-cell cross-sectional area.

### RNA sequencing analysis

Total RNA was extracted using the TRIzol reagent kit (Invitrogen) according to the manufacturer's protocol, followed by RNA quality assessment using an Agilent 2100 Bioanalyzer (Agilent). After total RNA was extracted, eukaryotic mRNA was enriched using Oligo (dT) beads, fragmented into short fragments, and reverse-transcribed into cDNA. The purified double-stranded cDNA fragments were end-repaired, had A bases added, and were ligated to Illumina sequencing adapters, after which the ligation reaction was purified and amplified through polymerase chain reaction (PCR). The resulting cDNA library was sequenced using an Illumina Novaseq6000.

Differential expression analysis of RNAs was performed utilizing DESeq between two different groups, and edgeR was used between two samples. Genes with a false discovery rate (FDR) < 0.05 and absolute fold change > 1 were considered differentially expressed genes (DEGs). GO and KEGG enrichment analyses were performed using DEGs to identify corresponding biological functions. GSEA was performed using GSEA and MSigDB to assess whether a set of genes in specific GO terms/KEGG pathways/Reactome pathways/DO terms showed significant differences between the two groups. Briefly, the gene expression matrix was input, and genes were ranked employing the SignaltoNoise normalization method. Enrichment scores and *p* value were calculated using the default parameters. Diagrams were plotted on online sites (OmicSmart and Xiantao Academic).

### Quantitative real-time polymerase chain reaction (RT-qPCR)

mRNA from CAR-T and mCAR-T cells was extracted using RNA Quick Purification Kit (ES Science) according to the manufacturer's instruction. Fast Reverse Transcription Kit (ES Science) was used for cDNA reverse transcription. The primers were purchased from Rui Biotech (Beijing, China), and all PCR reactions were performed on a LightCycler 480 (Roche) using Fast All-in-One RT Kit with SYBR Green Master Mix (ES Science).

### *In vivo* mouse models

Each NCG mouse was injected subcutaneously with 2×10^6^ CD133-SK-HEP-1 cells. Once tumors reached 50-100 mm^3^ in size, the mice were randomly divided into four groups. Subsequently, NT, CAR-T, and mCAR-T cells, totaling 1×10^7^ cells, were intravenously injected into the tumor-bearing mice. Tumor volume was measured using caliper and calculated with the following formula: tumor volume = (length×width^2^)/2. For the orthotopic mouse model construction, mice were anesthetized with pentobarbital sodium (75mg/kg) via intraperitoneal injection, followed by intrahepatic inoculation of 2 million CD133-SK-HEP-1-luc cells into the left liver lobe. After tumor formation, the mice were randomized, and 10 million NT, CAR-T, or mCAR-T cells were injected through the tail veins to treat tumor-bearing mice. Bioluminescence imaging was performed using a PerkinElmer IVIS imaging system with Living Image software to confirm tumor formation and monitor tumor growth after intraperitoneal injection of luciferin. Quantification for each mouse was recorded in photons per second.

### Statistical analysis

GraphPad Prism version 8.0.2 was used for data analysis and graph plotting. Comparisons between each two groups were made using two-tailed unpaired or paired *t* tests. For the statistics of CD3^+^ and Granzyme B^+^ cells in the tumors of the orthotopic mouse model, Mann-Whitney test was performed. Differences considered statistically significant are as follows: ns: not significant,* p* > 0.05; *p < 0.05; **p < 0.01; ***p < 0.001; and ****p < 0.0001.

## Results

### Long-term *ex vivo* expansion of T cells with metformin preserves their memory phenotype

**Figure 1:**
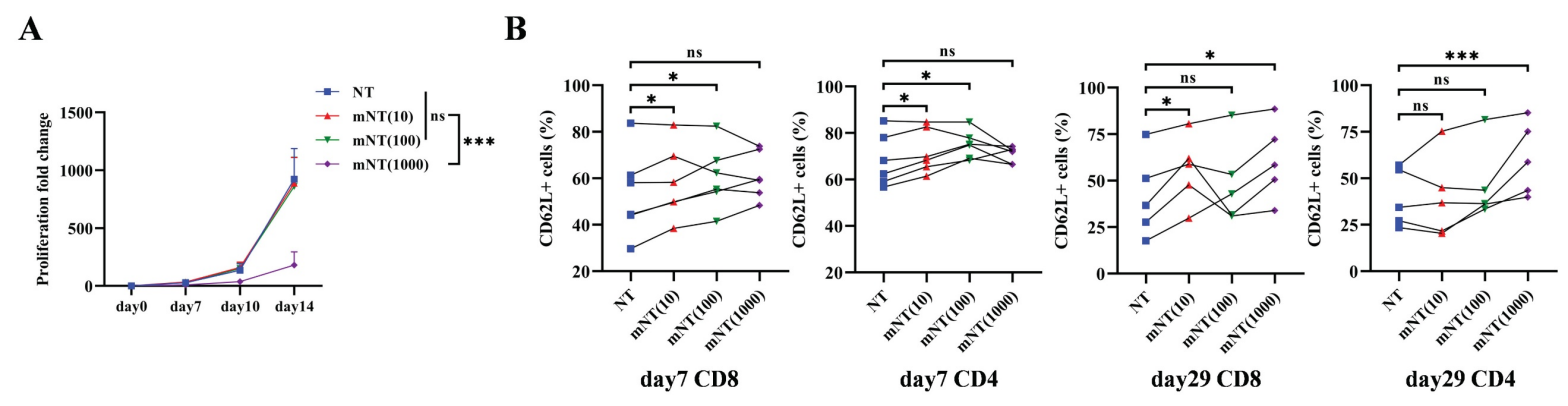
** T cells expanded in metformin shows increased memory phenotype.** (A) The proliferation fold change of each group from day 0 to day 14. Two-tailed paired *t*-tests (ns: not significant, *p* > 0.05; ***p < 0.001); mean ± SEM are shown (n = 5). (B) CD62L^+^ subsets in CD8 and CD4 T cells treated with or without different concentrations of metformin on day 7 (n = 6) and day 29 (n = 5). Two-tailed paired *t*-tests (ns: not significant, *p* > 0.05; *p < 0.05; ***p < 0.001). Data are shown as each independent sample.

A previous study reported that metformin treatment expanded the population of memory-like CXCR3^+^ CD8^+^ T cells against *Mycobacterium tuberculosis* infection in patients with type 2 diabetes^44^. To confirm whether metformin could sustain the T cell memory phenotype during *in vitro* culture, we first evaluated the impact of different concentrations of metformin on T cells from seven healthy donors. The proliferative capacity of T cells significantly decreased when metformin reached a concentration of 1000 μM, while no effect was noted with metformin concentrations of 10 μM and 100 μM (Figure 1A). Compared to un-transduced T (NT) cells without metformin treatment, NT cells treated with 10 μM and 100 μM metformin, referred to as mNT (10) and mNT (100) cells, respectively, exhibited a more memorized phenotype in both CD8^+^ and CD4^+^ T cells on day 7 (Figure 1B). Notably, mNT (10) cells maintained more memory subsets in CD8^+^ T cells on day 29 (Figure 1B), indicating that 10 μM metformin could sustain T cell memory over an extended period of time during *in vitro* culture. Unexpectedly, NT cells treated with 1000 μM metformin (mNT (1000)) also showed an increase in memory T cells compared to NT cells on day 29 (Figure 1B).

### CAR-T cells expanded with metformin maintain central memory phenotype and exhibit enhanced function

**Figure 2:**
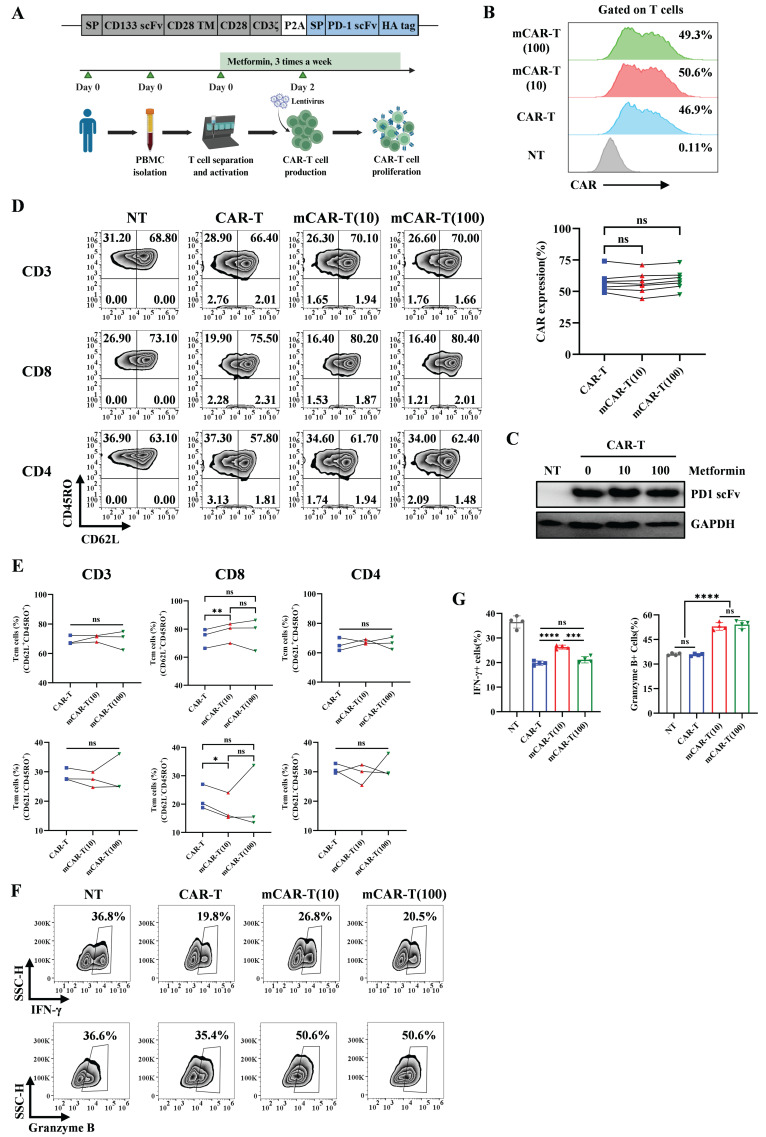
** Metformin-treated CAR-T cells maintain their central memory phenotype and have enhanced function.** (A) Upper: Schematic of PD1 scFv and CD133 CAR structure. Lower: Workflow of CAR-T cells production and expansion with metformin *in vitro*. (B) Upper: Representative flow cytometry plot showing the CAR expression in each group. Lower: The frequency of CAR^+^ T cells in each group from different healthy donors. Two-tailed paired *t*-tests (ns: not significant, *p* > 0.05). Data are shown as each independent sample (n = 7). (C) Western blot analysis of PD-1 blocking scFv measured via HA-tag in the concentrated culture supernatant of CAR-T cells treated with or without metformin. (D and E) Representative flow cytometry plots (D) and the frequency (E) of CAR-T cell memory subsets in CD3^+^, CD8^+^ and CD4^+^ T cells after treatment with or without different concentrations of metformin. Two-tailed paired *t*-tests (ns: not significant, *p* > 0.05; *p < 0.05; **p < 0.01). Data are shown as each independent sample (n = 3). (F and G) Representative (F) and quantitative (G) analysis of the percentages of IFN-γ^+^ and Granzyme B^+^ T cells in each group. Two-tailed unpaired *t*-tests (ns: not significant, *p* > 0.05; ***p < 0.001; ****p < 0.0001); mean ± SEM are shown (n = 4).

Our previous studies have demonstrated that PD-1 scFv-secreting CAR-T (PD1 scFv and CAR-T) cells have better antitumor effects than traditional CAR-T cells^10^. However, persistence remains a hurdle for CAR-T cells^45^-^47^. Current studies have shown that memory phenotype is important for the antitumor effects of CAR-T cells *in vivo*^48^-^52^. Data from NT and mNT cells indicated that a suitable concentration of metformin can maintain the memory T cell phenotype when expanding *in vitro*. To evaluate whether metformin could improve the ability of PD1 scFv and CAR-T cells by maintaining their memory phenotype, we constructed PD1 scFv-secreting and CD133-specific CAR-T (PD1 scFv and CD133 CAR-T, referred to as CAR-T) cells using a lentivirus approach based on our previous work^10^ (Figure 2A). The binding activity of the PD-1 blocking scFv secreted by CAR-T cells was validated in a dose-dependent manner by flow cytometry using PD-1-overexpressing T cells (Figure S1A and S1B). Given that 1000 μM metformin significantly inhibited T cell proliferation (Figure 1A), we excluded this concentration from subsequent experiments. In contrast, 10 μM and 100 μM metformin showed no effects on T cell proliferation (Figure 1A), CAR expression (Figure 2B), and PD-1 scFv secretion (Figure 2C). Therefore, we selected these two concentrations for further experiments.

We then evaluated the memory phenotype of CAR-T and mCAR-T cells. In mCAR-T (10) cells, we observed an increase in central memory T (Tcm, CD62L^+^ CD45RO^+^) cells and a concurrent decrease in effector memory T (Tem, CD62L^-^ CD45RO^+^) cells within the CD8^+^ CAR^+^ population, compared to conventional CAR-T cells, whereas these differences were not statistically significant within the CD4^+^ CAR^+^ population (Figure 2D and 2E). The function of T cells was also evaluated. We treated NT, CAR-T, mCAR-T (10) and mCAR-T (100) cells with BFA, monensin and PMA/ionomycin for 5 h, followed by flow cytometry. The result showed that mCAR-T (10) cells had both stronger IFN-γ and Granzyme B secreting ability, while mCAR-T (100) cells only secreted more Granzyme B compared with CAR-T cells (Figure 2F and 2G). For TNF-α secreting, there was no difference between CAR-T and mCAR-T cell groups (Figure S2A and S2B). These data suggested that *in vitro* culturing with metformin preserves the Tcm population among CAR-T cells and enhances their cytokine production capability.

### mCAR-T cells exhibit rapid and enhanced cytotoxicity and reduced exhaustion *in vitro*

To validate the specific cytotoxic effects of CAR-T and mCAR-T cells, we tested their cytotoxicity against CD133 high‑expression Hep3B and CD133-knockout (CD133-KO) Hep3B cells (Figure S3A). The results demonstrated that both CAR-T and mCAR-T cells exhibited effective cytotoxicity against wild-type Hep3B cells at an effector-to-target (E:T) ratio of 1:1 (Figure S3B), whereas showed markedly reduced cytotoxicity against CD133-KO Hep3B cells (Figure S3C), indicating the antigen specificity of CD133-specific CAR-T cells. Notably, mCAR-T (10) cells demonstrated stronger cytotoxicity than conventional CAR-T cells (Figure S3B).

**Figure 3:**
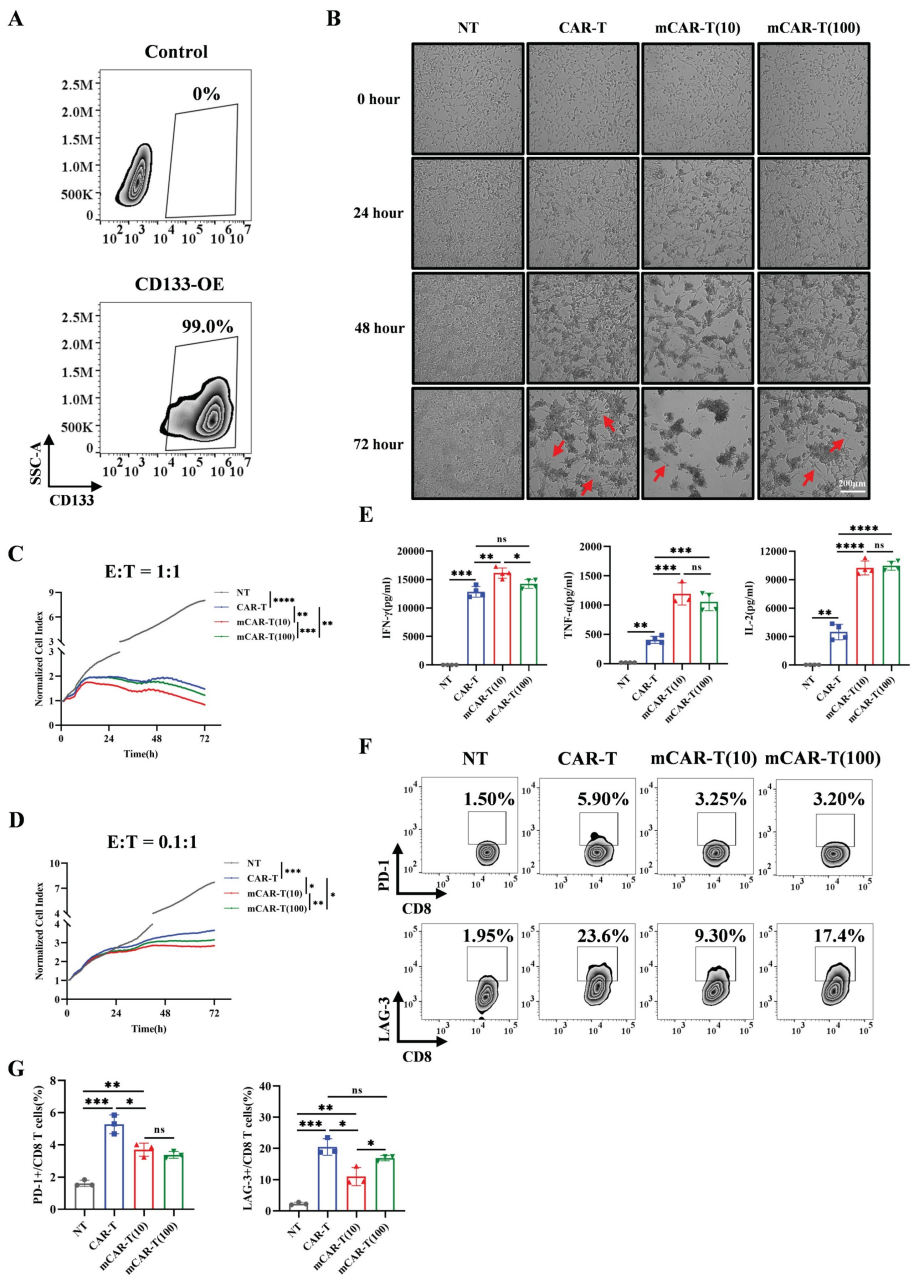
** mCAR-T cells exhibit enhanced cytotoxicity and reduced exhaustion after co-culture with target cells.** (A) CD133 expression on control and transduced SK-HEP-1 cells. (B) Representative images of NT, CAR-T, and mCAR-T cells against CD133-SK-HEP-1 cells for 24, 48 and 72 h at an E:T ratio of 1:1. Red arrows indicate residual tumor cells. (C and D) Normalized cell index values outputted continuously from the co-culture systems of NT, CAR-T, or mCAR-T cells and CD133-SK-HEP-1 cells at an E:T ratio of 1:1 (C) or 0.1:1 (D) for 72 h using xCELLigence RTCA system. The density changes of CD133-SK-HEP-1 cells after 72 h were calculated and compared using two-tailed unpaired *t*-tests (*p < 0.05; **p < 0.01; ***p < 0.001; ****p < 0.0001. n = 3). (E) Cytokine secretion of IFN-γ, TNF-α, and IL-2 in the supernatants of NT, CAR-T, and mCAR-T cells co-cultured with CD133-SK-HEP-1 cells at an E:T ratio of 1:1 for 48 h. Two-tailed unpaired *t*-tests (ns: not significant, *p* > 0.05; *p < 0.05; **p < 0.01; ***p < 0.001; ****p < 0.0001); mean ± SEM are shown (n = 4). (F and G) Representative (F) and quantitative (G) analysis of PD-1 and LAG-3 expression on NT, CAR-T, and mCAR-T cells co-cultured with CD133-SK-HEP-1 cells at an E:T ratio of 1:1 for 72 h. Two-tailed unpaired *t*-tests (ns: not significant, *p* > 0.05; *p < 0.05; **p < 0.01; ***p < 0.001); mean ± SEM are shown (n = 3).

We further constructed SK-HEP-1 cells overexpressing CD133 (CD133-SK-HEP-1 cells) (Figure 3A) and compared the cytotoxic capacities of CAR-T and mCAR-T cells. Both CAR-T and mCAR-T cells also exhibited continuous and specific cytotoxicity when encountered with CD133-SK-HEP-1 cells within 72 h at an E:T ratio of 1:1, and mCAR-T (10) cells demonstrated faster and stronger cytotoxicity compared to traditional CAR-T cells (Figure 3B, 3C and S3D), consistent with the results observed against Hep3B cells (Figure S3B). Further analysis revealed that at a low E:T ratio (0.1:1), mCAR-T cells still showed rapid and enhanced antitumor activity (Figure 3D and S3E). Notably, the advantages of mCAR-T cells were more pronounced at the low E:T ratio. After co-culturing for 72 h, the cytolysis percentage of mCAR-T (10) cells was 10.86% higher than that of CAR-T cells at an E:T ratio of 0.1:1 and only 8.62% higher when the E:T ratio was 1:1 (Figure S3F). These results showed that mCAR-T (10) cells exhibited the strongest cytotoxic activity against tumor cells among all groups. The cytokine-producing abilities of CAR-T and mCAR-T cells were evaluated following incubation with CD133-SK-HEP-1 cells. mCAR-T (10) cells showed stronger secretion of IFN-γ, TNF-α and IL-2 than that of conventional CAR-T cells at an E:T ratio of 1:1 after 48 h of co-culture (Figure 3E), which correlated with better cytokine production by mCAR-T (10) cells compared to traditional CAR-T cells when without target cell stimulation (Figure 2F and 2G). Cancer-induced CD8 T cell exhaustion has been well-researched, and many studies have aimed to achieve superior antitumor effects by restoring the function of exhausted CD8 T cells^53^-^56^. To further clarify whether metformin delays mCAR-T cell exhaustion when co-cultured with CD133-SK-HEP-1 cells, we detected inhibitory receptors on the surface of CD8^+^ T cells. The data indicated that mCAR-T (10) cells expressed lower levels of LAG-3 than CAR-T cells after 48 h of killing target cells (Figure S3G and S3H) and reduced expression of both PD-1 and LAG-3 after 72 h of co-culture (Figure 3F and 3G); conversely, there was no significant difference in the expression of TIM-3 between these two groups (Figure S3G-J).

Collectively, compared to CAR-T cells, mCAR-T (10) cells exhibited a rapid and stronger killing ability, accompanied by less inhibitory receptor expression against target cells.

### Transcription analysis reveals metformin-induced metabolism changes and memory phenotype promotion in CAR-T cells

**Figure 4:**
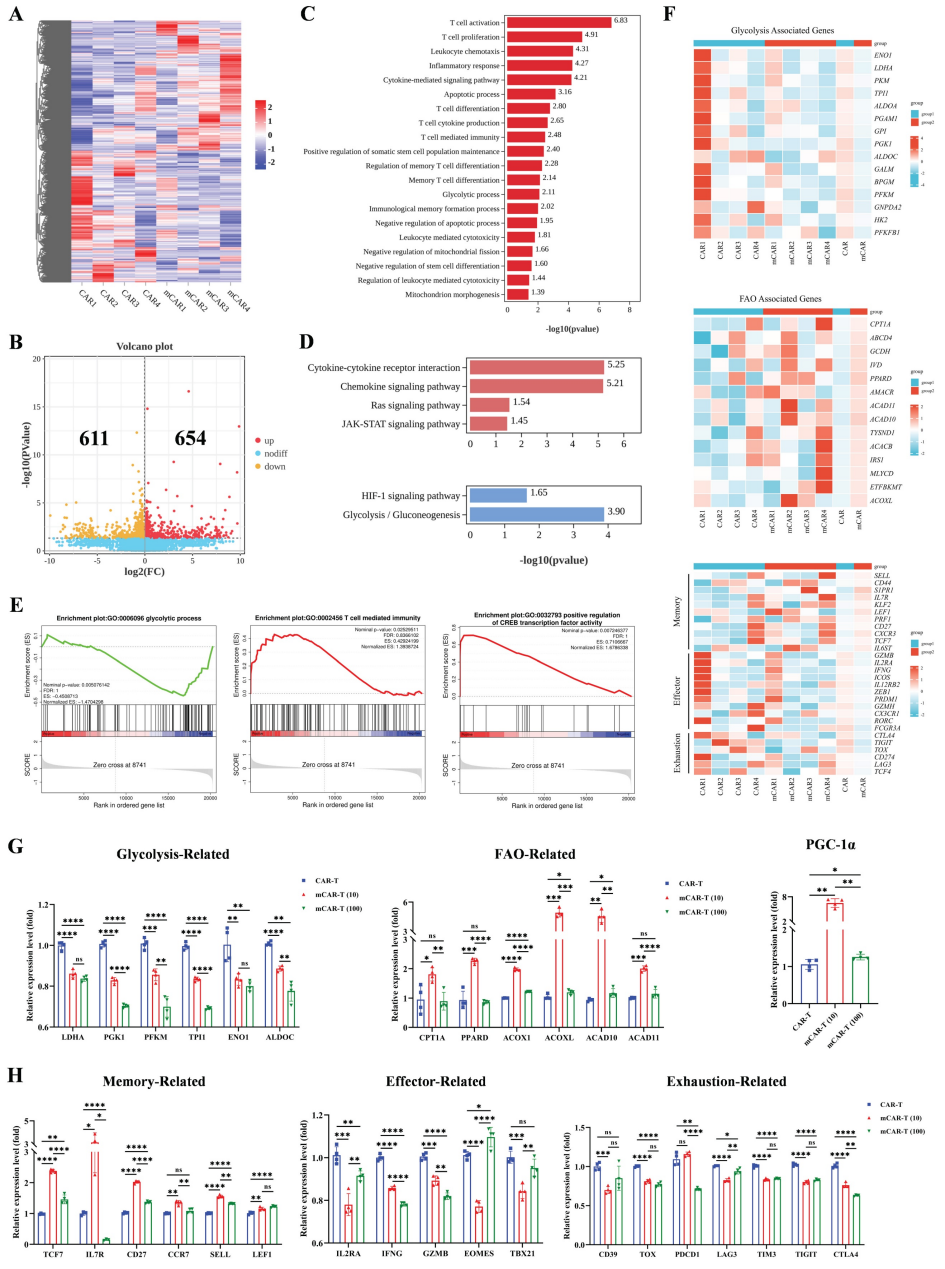
** Transcriptional characteristics of mCAR-T cells.** (A) Hierarchical clustering heatmap showing differentially expressed genes between mCAR-T cells and CAR-T cells (n = 4). (B) Volcano plot showing genes up- and downregulated in mCAR-T cells relative to traditional CAR-T cells (n = 4). (C) GO analysis for biological processes indicating the functional enrichment of differentially expressed genes in mCAR-T cells (n = 4). (D) Up- and downregulated signaling pathways in mCAR-T cells determined through KEGG analysis (n = 4). (E) Representative GSEA enrichment plots based on MSigDB demonstrating the up- and downregulated gene sets in mCAR-T cells (n = 4). (F) Heatmaps demonstrating the different expression profiles of glycolysis-, FAO-associated genes and T-cell memory-, effector- and exhaustion-related genes between CAR-T and mCAR-T cells (n = 4). (G) The expression levels of glycolysis- and FAO-related genes as well as PGC-1α in CAR-T and mCAR-T cells. Data are presented as mean ± SEM (n = 4), two-tailed unpaired *t*-tests (ns: not significant, p > 0.05; *p < 0.05; **p < 0.01; ***p < 0.001; ****p < 0.0001). (H) The expression levels of memory-, effector-, and exhaustion-related genes in CAR-T and mCAR-T cells. Data are presented as mean ± SEM (n = 3-4), two-tailed unpaired *t*-tests (ns: not significant, p > 0.05; *p < 0.05; **p < 0.01; ***p < 0.001; ****p < 0.0001).

To investigate the underlying mechanism by which metformin regulates the function and differentiation of mCAR-T cells, CAR-T and mCAR-T (10) cells from four healthy donors were treated with metformin for 14 d *in vitro* and subjected to bulk RNA sequencing. A heatmap showed differentially expressed genes (DEGs) between mCAR-T and CAR-T cells (Figure 4A). The volcano plot further revealed 654 and 611 upregulated and downregulated genes in mCAR-T cells compared to those in CAR-T cells (false discovery rate [FDR] < 0.05) (Figure 4B). Next, we performed Gene Ontology (GO) analysis and found that the DEGs were enriched in T cell activation, proliferation, differentiation, and apoptosis in the biological processes of mCAR-T cells (Figure 4C).

Kyoto Encyclopedia of Genes and Genomes (KEGG) analysis revealed that the Ras and JAK-signal transducer and activator of transcription (STAT) signaling pathways were upregulated in mCAR-T cells, whereas the HIF-1 signaling pathway and glycolysis/gluconeogenesis were downregulated (Figure 4D). Gene Set enrichment analysis (GSEA) further clarified the downregulation of glycolytic process-related genes and the upregulation of T cell-mediated immunity as well as cAMP-response element binding protein (CREB) transcription factor activity-related genes in mCAR-T cells (Figure 4E). To explore the differences in metabolism and differentiation between CAR-T and mCAR-T cells, heat maps were plotted based on representative genes. The data showed that glycolysis-associated genes were downregulated in mCAR-T cells, whereas fatty acid oxidation (FAO)-associated genes were upregulated (Figure 4F), which partly explains why mCAR-T cells had an increased Tcm cell subset^57^-^59^. Additionally, the mCAR-T cells exhibited enhanced expression of memory-related genes and reduced expression of effector- and exhaustion-related genes (Figure 4F).

To further validate the metformin-induced metabolic changes and memory-like phenotype in mCAR-T (10) cells revealed by RNA-seq analysis, quantitative real-time PCR was performed using specific primers (Table S1). The results demonstrated that compared to conventional CAR-T cells, in mCAR-T (10) cells, genes related to glycolysis (*LDHA*, *PGK1*, *PFKM*, *TPI1*, *ENO1*, and *ALDOC*) were significantly downregulated, while those associated with FAO (*CPT1A*, *PPARD*, *ACOX1*, *ACOXL*, *ACAD10*, and *ACAD11*) were markedly upregulated, and the transcription level of *PGC-1α*, the master regulator of mitochondrial biogenesis^19^, ^60^, ^61^, showed a significant increase (Figure 4G), consistent with the RNA-seq results. We also observed that the expression levels of representative genes associated with T-cell memory (*TCF7*, *IL7R*, *CD27*, *CCR7*, *SELL*, and *LEF1*) were significantly higher in mCAR-T (10) cells than in CAR-T cells, while the expression of genes related to effector function (*IL2RA*, *IFNG*, *GZMB*, *EOMES*, and *TBX21*) and exhaustion (*CD39*, *TOX*, *PDCD1*, *LAG3*, *TIM3*, *TIGIT*, and *CTLA4*) was markedly reduced (Figure 4H), further supporting our transcriptional findings.

Altogether, the results of transcription analysis and subsequent validation were in accordance with previous observations in our study and indicated that mCAR-T cells have enhanced oxidative metabolism and are less differentiated than conventional CAR-T cells.

### Promoting PGC-1α expression and STAT3 phosphorylation contribute to enhanced mitochondrial metabolism in CAR-T cells

**Figure 5:**
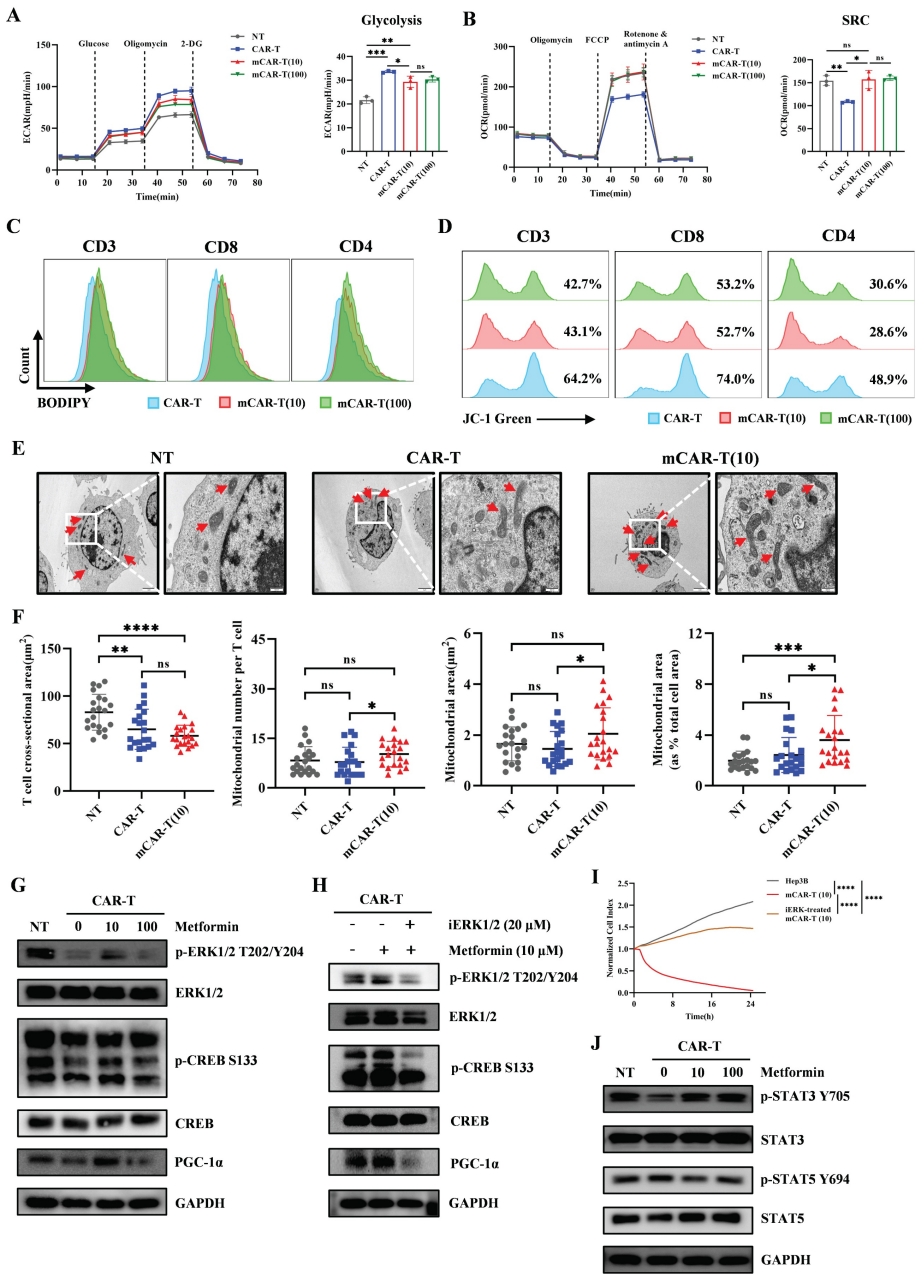
** Metformin enhances mitochondrial metabolism via PGC-1α and STAT3/STAT5 signaling.** (A) Left: The ECAR of NT, CAR-T, and mCAR-T cells measured employing Seahorse energy metabolism analyzer. Right: The statistical summary indicating glycolysis of each group. Two-tailed unpaired *t* test (ns: not significant, *p* > 0.05; *p < 0.05; **p < 0.01; ***p < 0.001); mean ± SEM are shown (n = 3). (B) Left: The OCR of NT, CAR-T, and mCAR-T cells measured utilizing Seahorse energy metabolism analyzer. Right: The SRC of each group. Two-tailed unpaired *t* test (ns: not significant, *p* > 0.05; *p < 0.05; **p < 0.01); mean ± SEM are shown (n = 3). (C) Representative flow cytometry plot of lipid content in CAR-T and mCAR-T cells detected through BODIPY 493/503. (D) Representative flow cytometry plot of MMP in CAR-T and mCAR-T groups detected via JC-1 green fluorescence. (E) Representative TEM images (2500× and 10000×) of NT, CAR-T, and mCAR-T cells. Red arrows indicate mitochondria. (F) T-cell size (shown as cross-sectional area) and mitochondrial morphology quantification (mitochondrial number, mitochondrial cross-sectional area, and mitochondrial cross-sectional area relative to total T-cell cross-sectional area) in NT, CAR-T, and mCAR-T cells. Two-tailed unpaired *t* test (ns: not significant, *p* > 0.05; *p < 0.05; **p < 0.01; ***p < 0.001; ****p < 0.0001); mean ± SEM are shown (n = 21). (G) Western blot analysis of ERK1/2, pERK1/1, CREB, pCREB, and PGC-1α levels in NT, CAR-T, and mCAR-T cells. (H) Western blot analysis of the expression levels of the ERK/CREB/PGC-1α axis in CAR-T, mCAR-T (10), and iERK-treated mCAR-T (10) cells. (I) The cytotoxicity of mCAR-T (10) cells with or without iERK treatment against Hep3B cells at an E:T ratio of 1:1. Data are presented as the mean of replicates (n = 3), two-tailed unpaired *t*-tests (****p < 0.0001). (J) Western blot analysis of STAT3, pSTAT3, STAT5, and pSTAT5 levels in NT, CAR-T, and mCAR-T cells.

To examine how metformin treatment improves the metabolic fitness in mCAR-T cells, we conducted the seahorse metabolic flux assay and observed that mCAR-T cells demonstrated lower extracellular acidification rate (ECAR) and glycolysis, along with higher potential in oxygen consumption rate (OCR) and mitochondrial spare respiratory capacity (SRC) (Figure 5A and 5B). These findings indicate that mCAR-T cells have developed a metabolic profile with increased capacity for OXPHOS for energy production, which is crucial for differentiation into memory T cells^62^, ^63^. In contrast, traditional CAR-T cells rely more on glycolysis for energy production. However, after co-culture with CD133-SK-HEP-1 cells for 12 h, mCAR-T (10) cells showed enhanced glycolysis and OXPHOS compared to traditional CAR-T cells (Figure S4B and S4C). Interestingly, although mCAR-T and CAR-T cells exhibited the same level of basal OCR (Figure S4A), after antigen stimulation, mCAR-T (10) cells showed a higher basal OCR than CAR-T cells (Figure S4C). We also measured the lipid content of the cells using BODIPY 493/503. mCAR-T cells exhibited stronger lipid uptake capacity in both CD4^+^ and CD8^+^ T cells (Figure 5C). Alterations in mCAR-T cell metabolism prompted a deeper study of cellular mitochondrial status, which is closely related to oxidative metabolism^16^, ^18^, ^62^. We first used JC-1 dye to detect mitochondrial membrane potential (MMP). In both CD4^+^ and CD8^+^ T cells, mCAR-T cells showed much less green fluorescence than CAR-T cells (Figure 5D), indicating that metformin treatment reduced MMP depolarization and thus inhibited the apoptosis of mCAR-T cells. Transmission electron microscope (TEM) was also used to visualize the mitochondrial content in NT, CAR-T and mCAR-T (10) cells (Figure 5E). mCAR-T cells showed an increase in the number of mitochondria and mitochondrial cross-sectional area compared to CAR-T cells (Figure 5F). Notably, there was a 1.49-fold increase in the mitochondrial cross-sectional area relative to the total T-cell cross-sectional area in mCAR-T cells, indicating an 182% increase in the mitochondrial volume for mCAR-T cells relative to control CAR-T cells (Figure 5F).

Previous bulk RNA sequencing suggested that CAR-T cells treated with 10μM metformin showed increased activity in the JAK-STAT and Ras signaling pathway (Figure 4D), prompting us to explore the phosphorylation of STAT family and ERK1/2 (downstream protein of Ras pathway) in CAR-T and mCAR-T cells. As expected, mCAR-T (10) cells exhibited higher levels of p-ERK1/2, leading to activation of the transcription factor CREB and increased CREB phosphorylation, as well as enhanced PGC-1α expression (Figure 5G). This is vital for mitochondrial biogenesis^19^, ^60^, ^61^, consistent with the observed increase in mitochondrial content and enhanced mitochondrial metabolism of mCAR-T (10) cells. To further explore the role of the ERK/CREB/PGC-1α axis in metformin-mediated enhancement of CAR-T cell function, we first performed western blot analysis to verify that ERK inhibitor (iERK) treatment could effectively suppress the activation of this axis (Figure 5H), and then conducted cytotoxicity assays using mCAR-T (10) cells with or without iERK treatment. The results showed that iERK-treated mCAR-T (10) cells exhibited significantly impaired cytotoxicity against Hep3B cells (Figure 5I), indicating that blocking the ERK/CREB/PGC-1α axis abolishes metformin's function on CAR-T cells.

Current studies have shown that STAT3 induces FAO and plays a crucial role in T cell memory formation, whereas STAT5 promotes T cell terminal differentiation and suppresses memory formation^64^-^66^. Consistently, western blot analysis showed increased STAT3 and decreased STAT5 phosphorylation in mCAR-T cells (Figure 5J), partly explaining the increased lipid content and Tcm cell subset in mCAR-T cells compared to those in traditional CAR-T cells.

In brief, metformin promotes mitochondrial biogenesis by activating the ERK/CREB/PGC-1α axis, and simultaneously activates STAT3, while inhibits STAT5 phosphorylation, thereby reprogramming CAR-T cell metabolism* in vitro*.

### Improved metabolic fitness and central memory induced *in vitro* translates into enhanced antitumor activity *in vivo*

**Figure 6:**
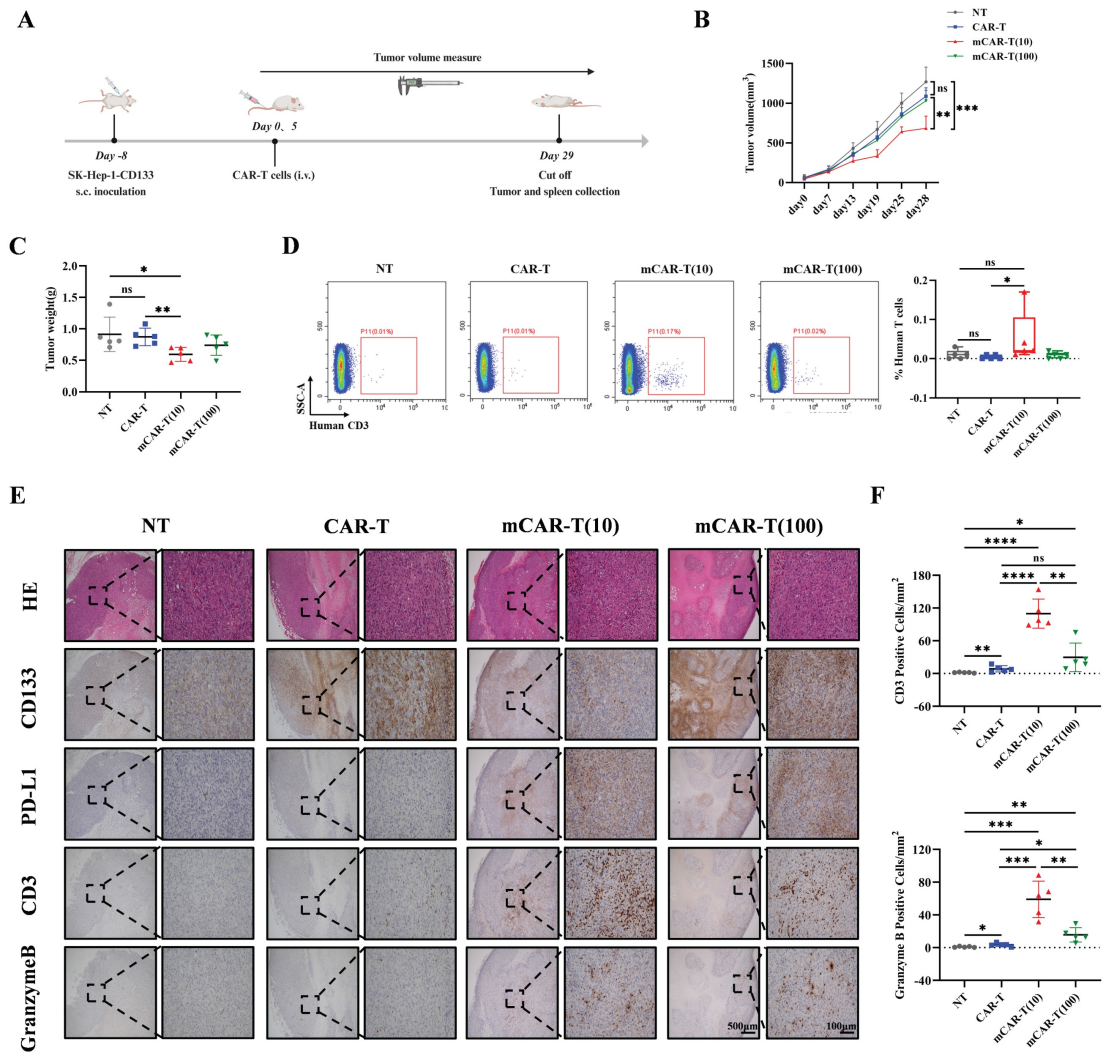
** mCAR-T cells exhibit enhanced antitumor activity in CD133-SK-HEP-1 cell subcutaneous xenograft model.** (A) Experimental schematic of an *in vivo* model testing the antitumor activity of CAR-T and mCAR-T cells against the CD133-SK-HEP-1 cell subcutaneous (s.c.) xenograft mouse model. (B) Tumor volume was monitored through caliper measurement over time and calculated and compared at the end of the experiment using two-tailed unpaired *t* test (ns: not significant, *p* > 0.05; **p < 0.01; ***p < 0.001); mean ± SEM are shown (n = 5). (C) Tumor weight of different treatment groups at the end of the experiment. Two-tailed unpaired *t* tests (ns: not significant, *p* > 0.05; *p < 0.05 and **p < 0.01); mean ± SEM are shown (n = 5). (D) Representative flow cytometry profile (left) and statistical box plot(right) showing the frequencies of human T cells in mouse spleens in different groups. Two-tailed unpaired *t* tests (ns: not significant, *p* > 0.05; *p < 0.05); mean ± SEM are shown (n = 5). (E) Representative HE and IHC staining images (40× and 200×) of CD133, PD-L1, CD3, and Granzyme B in each group. (F) The number of CD3^+^ and Granzyme B^+^ cells per mm^2^ of tumor cross-sectional area in each group analyzed utilizing Digital HALO Software. Two-tailed unpaired *t* tests (ns: not significant, *p* > 0.05; *p < 0.05; **p < 0.01; ***p < 0.001; ****p < 0.0001); mean ± SEM are shown (n = 5).

To explore whether the improved metabolic fitness of mCAR-T cells *in vitro* could translate into enhanced antitumor activity *in vivo*, 2 million CD133-SK-HEP-1 cells were subcutaneously inoculated into NCG mice. On days 8 and 13 after inoculation, a total of 1×10^7^ NT, CAR-T, mCAR-T (10) or mCAR-T (100) cells were intravenously injected to treat tumor-bearing mice (Figure 6A). The tumor volume in the mCAR-T (10) group was significantly lower than that in the CAR-T group on day 28 (Figure 6B). At the end of the experiment, the tumor weight in the mCAR-T (10) cell-treated group were much smaller than those in the CAR-T cell-treated group (Figure 6C). Human T cells in the mouse spleen were also tested. Unlike the other groups, which had almost no detectable human T cells, mice in the mCAR-T (10) cell-treated group had many more human T cells in their spleens (Figure 6D). This indicates that mCAR-T (10) cell treatment may induce systemic immunity in mice, which is significant for long-term tumor suppression^67^-^69^. Furthermore, we performed immunohistochemistry (IHC) staining to observe T cell infiltration and evaluate their functions. We classified the IHC staining slides (Figure S5A) and counted the CD3^+^ and Granzyme B^+^ cells in the tumor area. The mCAR-T (10) cell-treated group had more CD3^+^ and Granzyme B^+^ T cells inside the tumors (Figure 6E and 6F), consistent with the superior antitumor efficacy of mCAR-T (10) cells. Intriguingly, CD133 staining revealed lower CD133 expression in the mCAR-T (10) cell-treated group than in the CAR-T cell-treated group, indicating that CD133 protein may be suppressed by mCAR-T (10) cells (Figure 6E). The expression of PD-L1 increased in mCAR-T (10) and mCAR-T (100) cell-treated group, which might be attributed to increased IFN-γ secretion in mCAR-T cells^70^-^72^ (Figure 6E). Increased PD-L1 expression in tumors also indicated the rationality of constructing PD-1 secreting CAR-T cells in our previous study.

**Figure 7:**
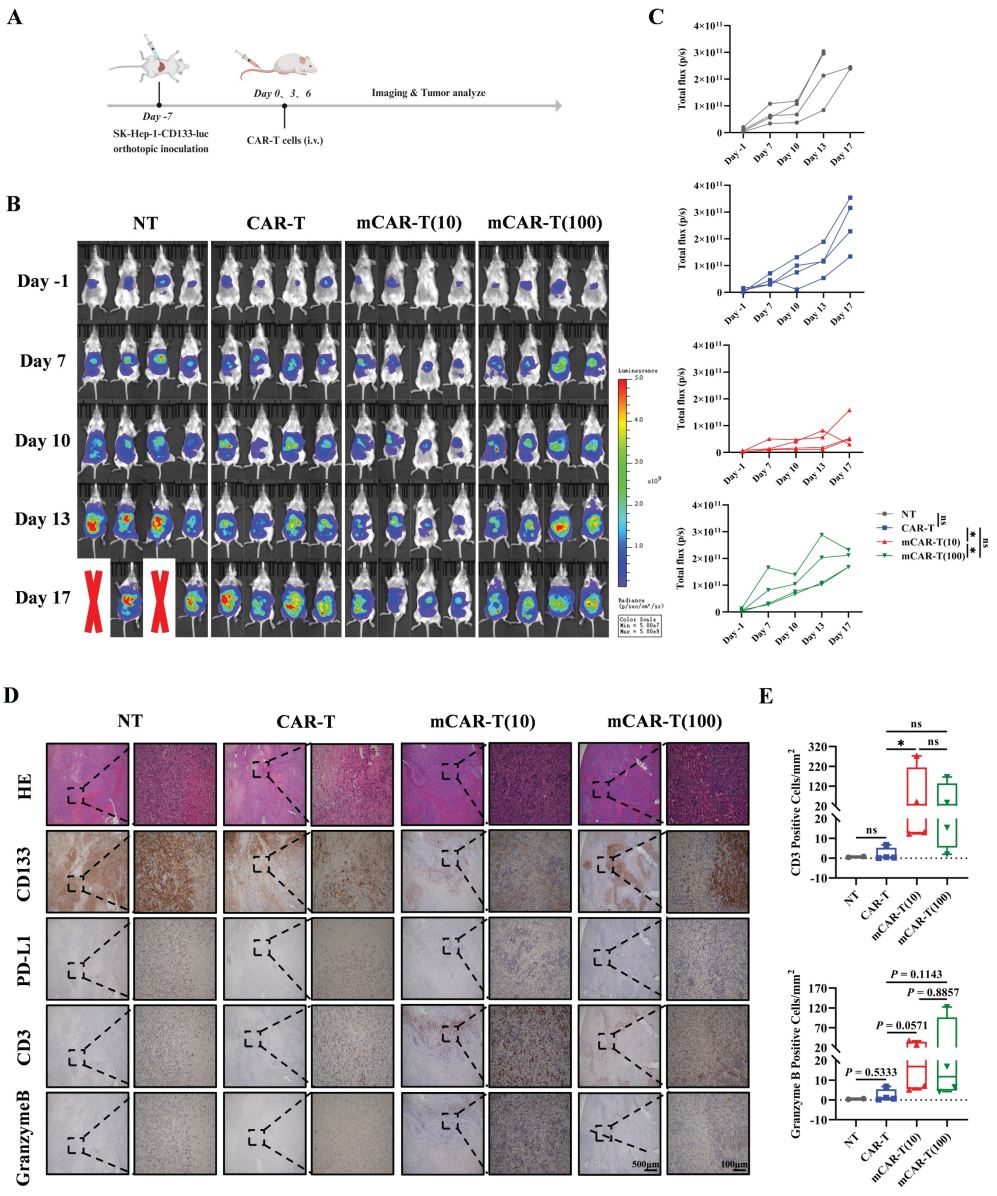
** mCAR-T cells show improved antitumor ability in orthotopic mouse model of HCC.** (A) Experimental schematic of another *in vivo* model to test the antitumor effects of CAR-T and mCAR-T cells against the CD133-SK-HEP-1-luc cell orthotopic mouse model. (B) BLI of tumor growth at different time points in each group (n = 4). Red X symbols indicate death of mice during the experiment. (C) Tumor growth curves of each treatment group determined by total bioluminescence flux in Figure 7B. The total bioluminescence flux on day 17 in different groups was calculated and compared using two-tailed unpaired *t* test (ns: not significant, *p* > 0.05; *p < 0.05). Data are shown as each independent sample (n = 4). (D) Representative HE and IHC staining images (40× and 200×) of CD133, PD-L1, CD3, and Granzyme B in each group. (E) Box plots indicating the number of CD3^+^ and Granzyme B^+^ cells per mm^2^ of tumor cross-sectional area in each group analyzed using Digital HALO Software. Mann-Whitney test (ns: not significant, *p* > 0.05; *p < 0.05). Mean ± SEM are shown (n = 4).

Next, to better simulate the real situation *in vivo*, an orthotopic mouse model of HCC was constructed by intrahepatic inoculation of 2 million CD133-SK-HEP-1 cells transduced to stably express luciferase (CD133-SK-HEP-1-luc cells), and 1×10^7^ NT, CAR-T, mCAR-T (10) or mCAR-T (100) cells were injected intravenously on days 7, 10 and 13 after inoculation (Figure 7A). Tumor growth was monitored via bioluminescence imaging (BLI), and mice treated with mCAR-T (10) cells had a significantly lower tumor burden than those treated with CAR-T and mCAR-T (100) cells (Figure 7B and 7C). Next, the classification (Figure S5B) and statistical analysis of IHC staining slides were performed as previously described. Similar to the results observed in the subcutaneous xenograft model, the mCAR-T (10) group had more CD3^+^ and Granzyme B^+^ cells in the tumors, reduced CD133 expression, and increased PD-L1 expression in the tumor cells compared to the CAR-T group (Figure 7D and 7E). In conclusion, CAR-T cells cultured with 10 μM metformin demonstrate greater expansion, persistence, and infiltration into the tumor *in vivo*, thereby enhancing their antitumor ability.

### Relapsed HCC patients may benefit from CD133-specific CAR-T cell therapy

**Figure 8:**
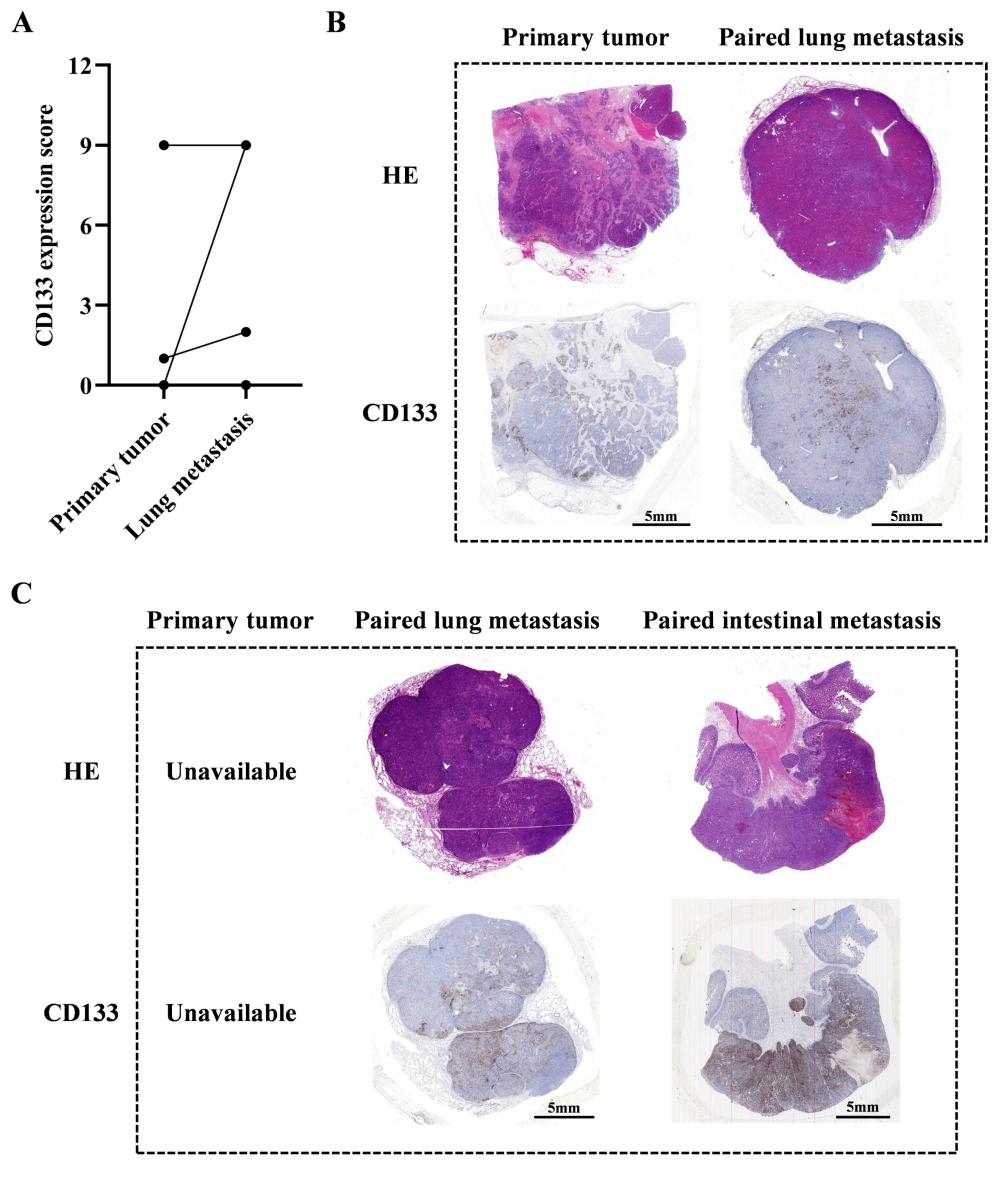
** The evaluation of CD133 expression of paired primary and metastatic tumors in HCC patients.** (A) The statistical summary indicating CD133 expression in primary and lung metastatic tumors in 8 paired samples (P001 to P008), except patient P009 whose primary tumor could not be available. (B) The HE and IHC staining images of CD133 in primary and lung metastatic tumors of patient P007. (C) The HE and IHC staining images of CD133 in lung and intestinal metastatic tumors of patient P009.

Our previous study indicates that male patients with advanced HCC could potentially benefit from CD133-specific CAR-T cell therapy^10^. In this study, we further collected 9 paired primary and metastatic tumor samples to evaluate their CD133 expression levels (Table S2). Our results indicated that lung metastatic tumors exhibited higher CD133 expression than primary tumors in 3 paired samples (P004, P005 and P008). In patient P007, both primary and lung metastatic tumors had high CD133 expression. In the remaining 4 paired samples (P001, P002, P003 and P006), neither primary nor lung metastatic tumors expressed CD133 (Figure 8A and 8B). Additionally, we observed high CD133 expression in lung and intestinal metastatic tumors of patient P009 (the primary tumor was unavailable) (Figure 8C). These data suggest that CD133-specific CAR-T cell therapy may be promising for those who have undergone conventional therapies and exhibit high CD133 expression in their primary or metastatic tumors. For these patients, our PD1 scFv and CD133 CAR-T cells may be a suitable therapeutic strategy, potentially enhanced by metformin treatment *in vitro* during CAR-T cell manufacture.

## Discussion

**Figure 9:**
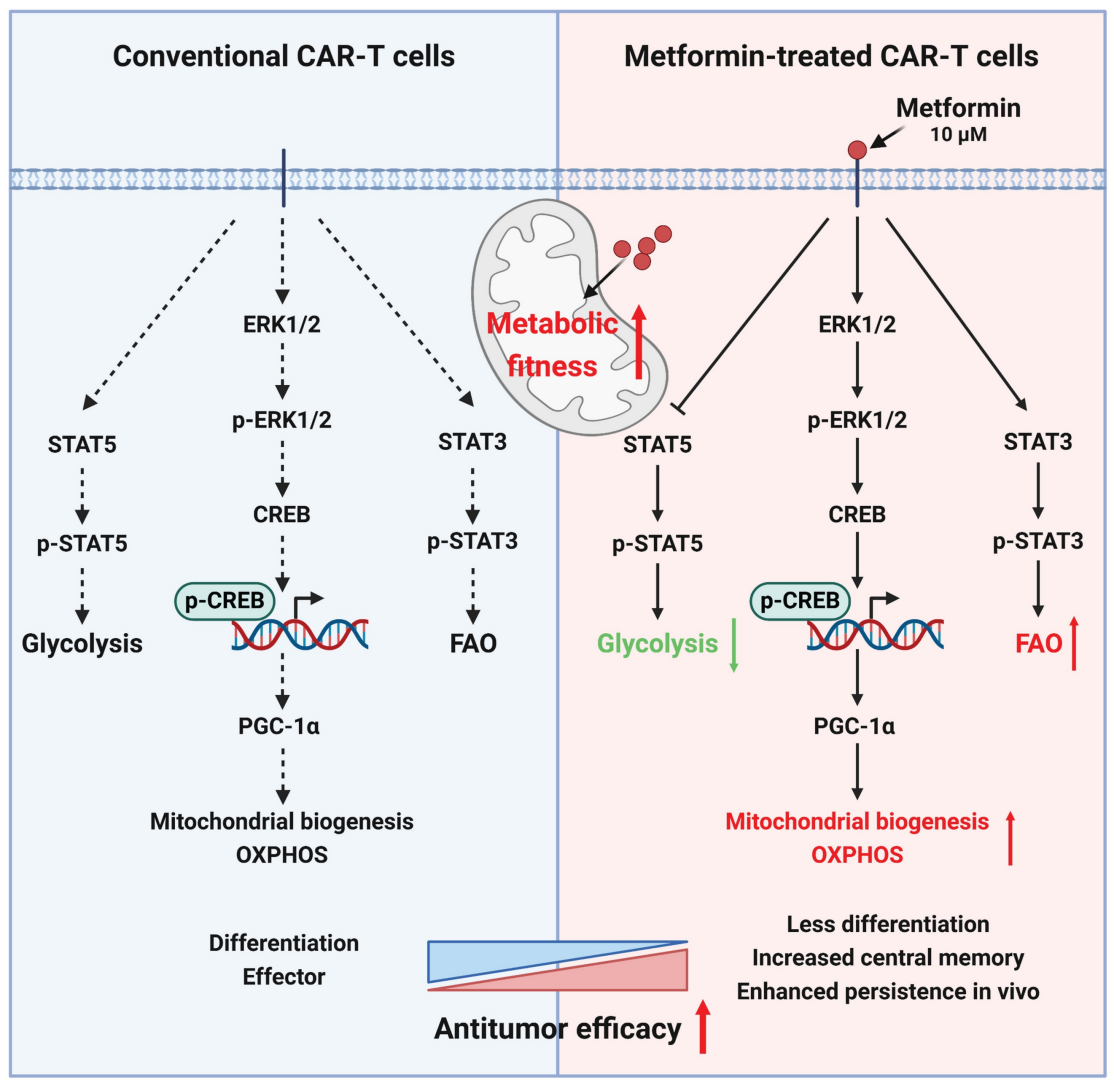
Mechanisms for the enhanced antitumor ability of metformin-treated CAR-T cells.

The persistence of CAR-T cells is crucial for their efficacy in addressing solid tumors^73^-^75^. Our investigation, utilizing *in vitro* cytotoxicity analyses, RNA sequencing, and subcutaneous and in situ models of liver cancer, revealed that, compared to untreated CAR-T cells, CAR-T cells treated with an appropriate concentration (10 μM) of metformin *in vitro* exhibited a superior antitumor effect *in vivo* by enhancing metabolic fitness via activating ERK/CREB/PGC-1α axis and promoting STAT3 while inhibiting STAT5 phosphorylation (Figure 9).

The maintenance of the memory phenotype is pivotal for the CAR-T cell expansion within the hostile TME of solid tumors. Previous studies have highlighted a memory phenotype *in vitro* that correlates with enhanced treatment effects *in vivo*, as evidenced by preclinical and clinical trials^36^, ^74^, ^76^-^78^. A previous study reported that metformin could induce a memory phenotype in CD8^+^ T cells in patients with type 2 diabetes. Metformin treatment promotes the expansion of a memory-like population of antigen-inexperienced CD8^+^ CXCR3^+^ T cells, demonstrating additional antimycobacterial properties. Notably, this response was absent in Cxcr3^-/-^ mice because CXCR3 deficiency impairs the generation of memory-like T cell populations mediated by FOXO1^44^. Consistent with this finding, our study also found that metformin influenced the memory phenotype of CAR-T cells, particularly CD8^+^ CAR-T cells, but had no significant effect on CD4^+^ CAR-T cells.

Herein, metformin-treated CAR-T cells showed a significant increase in mitochondrial numbers and mitochondrial area (% total T-cell area) compared to conventional CAR-T cells. This suggests that metformin treatment promotes mitochondrial biogenesis in T cells, leading to enhanced SRC and reduced glycolysis, thereby supporting the maintenance of a memory phenotype. Bharath et al. reported that metformin can lower age-related inflammation, enhance autophagy levels in CD4^+^ T cells in older patients, and alter mitochondrial bioenergetics and T cell inflammatory characteristics to levels comparable to those in younger patients^32^. Unlike the above study, our research indicated that metformin promoted mitochondrial biogenesis and improved mitochondrial function in CAR-T cells through ERK/CREB/PGC-1α axis. Recently, studies have reported that the enforced PGC-1α expression favors CD8^+^ T cell central memory formation. Tumor-infiltrating lymphocytes and CAR-T cells that overexpress PGC-1α exhibit enhanced antitumor capacity in mouse models^60^, ^79^. Our study demonstrated that metformin may improve or maintain the metabolic fitness of CAR-T cells through promoting mitochondrial biogenesis by increasing PGC-1α expression, which could contribute to their *in vivo* persistence.

The STAT proteins play a pivotal role in orchestrating the highly dynamic metabolism, regulating the mitochondrial activity at various levels, including energy metabolism and lipid-mediated mitochondrial integrity^80^. Previous reports have highlighted that STAT3 in breast tumor-associated CD8^+^ effector T cells promote FAO activity by upregulating CPT1B. Interestingly, the activation of STAT3 by leptin, particularly in association with increased adipose tissue, inhibits glycolysis and IFN-γ secreting in CD8^+^ T effector cells. Blocking the leptin-STAT3-FAO pathway reactivates breast tumor CD8^+^ T cells^65^. Additionally, this study demonstrated that PD1-induced FAO is partly mediated by STAT3 in tumor-associated T cells^65^. *In vivo* STAT3 activation enhances FAO and inhibits glycolysis in CD8^+^ T effector cells, impairs their effector function, and promotes tumor growth^65^, but *in vitro* STAT3 activation leads to a stronger antitumor ability *in vivo* because of T cell memory formation, which is crucial for long-term antitumor immune responses. Conversely, unlike STAT3, activation of FAO, STAT5, an important regulator of glucose uptake in T cells^81^, mediates glycolysis, induces T cell exhaustion, and hampers memory T cell stemness. In this study, we observed enhanced STAT3- and decreased STAT5-phosphorylation in metformin-treated CAR-T cells, which correlated with enhanced lipid uptake and reduced glycolysis. This led to an increased Tcm cell population *in vitro*, consistent with prior reports^64^, ^65^, ^81^. In particular, the efficacy of adoptive T cell or NK cell therapy may benefit from the presence of intact or even increased STAT3 function. Targeting STAT3 *in vitro* may enhance the therapeutic effects of CAR-T cells.

The present study still has several limitations. First, only one CAR-T cell model was used for the analysis. Therefore, it is necessary to explore whether metformin can enhance the antitumor ability in other CAR-T cell models. Although recently other studies have shown that metformin could improve the function of γδ T cells^82^ or CEA CAR-T cells^83^, it has also been reported that CD19 CAR-T cells treated with high concentrations (1, 10, and 20 mM *in vitro*, and 5 mg/mL *in vivo*) of metformin exhibit a worse antitumor efficacy^84^, and different CAR scFvs cluster may induce early exhaustion and limit antitumor efficacy of CAR-T cells^85^. Moreover, different costimulatory domains need to be explored. 4-1BB co-stimulation is always considered a better choice because of its healthy condition and mitochondrial fitness compared to CD28 in CAR-T cells^86^. Second, PGC-1α and STAT3 may be crucial factors contributing to the enhancement of metformin-treated CAR-T cells compared to non-treated cells. It is worthwhile to explore whether blocking the ERK/CREB/PGC-1α axis or inhibiting STAT3 would abolish or maintain the enhancing effects of metformin on CAR-T cells. Third, influencing the culture system to improve the antitumor ability of CAR-T cells deserves more in-depth consideration. Decreasing glycolysis and enhancing FAO to promote a memory phenotype during CAR-T cell expansion represents a promising direction. However, addressing the duration of these effects in long-term *in vitro* culture remains a challenge. Studies have reported that preconditioning CAR-T cells with drugs can enhance their infiltration into solid tumor^24^, ^76^. Some approaches focus on maintaining optimal drug concentrations *in vivo* to maximize effectiveness^87^, ^88^. Addressing gaps in understanding, particularly regarding mechanistic insights, should be prioritized in future research efforts^81^.

In conclusion, our study highlights that CAR-T cell manufacturing *in vitro* provides a valuable window for intervention. *In vitro* metformin treatment enhances CAR-T cell antitumor activities by improving mitochondrial function and maintaining memory phenotype via increasing PGC-1α expression, and promoting STAT3 while inhibiting STAT5 signaling. The use of PD-1 scFv-secreting and CD133-specific CAR-T cells treated with metformin *in vitro* could be an effective approach for treating relapsed HCC patients. This study provides a theoretical basis for its clinical application.

## Supplementary Material

Supplementary figures and table.

## Abbreviations

BLI: bioluminescence imaging
CAR-T: chimeric antigen receptor T-cell
CREB: cAMP-response element binding protein
DEGs: differentially expressed genes
DMEM: Dulbecco's modified Eagle's medium
ECAR: extracellular acidification rate
FAO: fatty acid oxidation
FBS: fetal bovine serum
FDR: false discovery rate
GO: Gene Ontology
GSEA: Gene Set enrichment analysis
HCC: hepatocellular carcinoma
IHC: immunohistochemistry
KEGG: Kyoto Encyclopedia of Genes and Genomes
mCAR-T: CAR-T cells treated with metformin
MMP: mitochondrial membrane potential
mNT: NT cells treated with metformin
NT: un-transduced T-cell
OCR: oxygen consumption rate
OXPHOS: oxidative phosphorylation
PGC-1α: peroxisome proliferator-activated receptor gamma coactivator-1alpha
SRC: spare respiratory capacity
STAT: signal transducer and activator of transcription
STR: short tandem repeat
Tcm: central memory T-cell
Tem: effector memory T-cell
TME: tumor microenvironment
