# Supplementary material for: Enhancing CAR-T Cell Metabolic Fitness and Memory Phenotype for Improved Efficacy against Hepatocellular Carcinoma
Source: Int J Biol Sci. 2025 Jun 20;21(9):4231–51. doi: 10.7150/ijbs.110406 (PMC12224003; doi:10.7150/ijbs.110406)
Supplement: Supplementary file 1 — Supplementary figures and table. [file ijbsv21p4231s1.pdf]

Supplementary information

Enhancing CAR-T Cell Metabolic Fitness and Memory Phenotype for Improved Efficacy against Hepatocellular Carcinoma

Jinqi You, Xinyi Yang, Jingjing Zhao, Hao Chen, Yan Tang, Dijun Ouyang, Yuanyuan Liu, Yan Wang, Songzuo Xie, Yuanyuan Chen, Jinghao Liao, Tong Xiang, Jianchuan Xia, Chaopin Yang and Desheng Weng

Supplementary table 2

Supplementary figures and figure legends 5

Supplementary table

Table S1. Primer sequences used for RT-qPCR

Gene Name	Forward Primer (5′→3′)	Reverse Primer (5′→3′)
LDHA	ATGGCAACTCTAAAGGATCAGC	CCAACCCCAACAACTGTAATCT
PGK1	GAACAAGGTTAAAGCCGAGCC	GTGGCAGATTGACTCCTACCA
PFKM	AGCGTTTCGATGATGCTTCAG	GGAGTCGTCCTTCTCGTTCC
TPI1	ACTGCCTATATCGACTTCGCC	AAGCCCCATTAGTCACTTTGTAG
ENO1	GCCGTGAACGAGAAGTCCTG	ACGCCTGAAGAGACTCGGT
ALDOC	GCCAAATTGGGGTGGAAAACA	TTCACACGGTCATCAGCACTG
CPT1A	TCCAGTTGGCTTATCGTGGTG	TCCAGAGTCCGATTGATTTTTGC
PPARD	GCCTCTATCGTCAACAAGGAC	GCAATGAATAGGGCCAGGTC
ACOX1	ACTCGCAGCCAGCGTTATG	AGGGTCAGCGATGCCAAAC
ACOXL	ATTGGCTATTTGGTGGTGCTATC	TCTTCTCCGCATTTTCACACG
ACAD10	GTACGAACCTGGGTTAAGCAG	CAGCCATTCGATCAGCCTCT
ACAD11	TTGGATTCCCCGTTCCCAAG	AAATCACGGAAGATTCGACCC
PGC-1α	TCTGAGTCTGTATGGAGTGACAT	CCAAGTCGTTCACATCTAGTTCA
TCF7	TTGATGCTAGGTTCTGGTGTACC	CCTTGGACTCTGCTTGTGTC
IL7R	CTCCAACCGGCAGCAATGTAT	AGATGACCAACAGAGCGACAG
CD27	TGCAGAGCCTTGTCGTTACAG	GCTCCGGTTTTCGGTAATCCT
CCR7	AAGCGATGCGATGCTCTCTC	TTGCGCTCAAAGTTGCGTG
SELL	ACCCAGAGGGACTTATGGAAC	GCAGAATCTTCTAGCCCTTTGC
LEF1	TGCCAAATATGAATAACGACCCA	GAGAAAAGTGCTCGTCACTGT
IL2RA	GAACACAACGAAACAAGTGACAC	GGCTGCATTGGACTTTGCATT
IFNG	TCGGTAACTGACTTGAATGTCCA	TCGCTTCCCTGTTTTAGCTGC
GZMB	CCCTGGGAAAACACTCACACA	GCACAACTCAATGGTACTGTCG
EOMES	CTGCCCACTACAATGTGTTCG	GCGCCTTTGTTATTGGTGAGTTT
TBX21	TTGAGGTGAACGACGGAGAG	CCAAGGAATTGACAGTTGGGT
CD39	TTGGAGCTTTGGACCTTGGG	TTATCTGGGGACTCGATAGTCTG
TOX	GTGATGCCAGATATACGAAACCC	AGCTGTGACTGGTTAATGGTAGT

PDCD1	CCAGGATGGTTCTTAGACTCCC	TTTAGCACGAAGCTCTCCGAT
LAG3	GCCTCCGACTGGGTCATTTT	CTTTCCGCTAAGTGGTGATGG
TIM3	TTGGACATCCAGATACTGGCT	CACTGTCTGCTAGAGTCACATTC
TIGIT	TCTGCATCTATCACACCTACCC	CCACCACGATGACTGCTGT
CTLA4	CATGATGGGGAATGAGTTGACC	TCAGTCCTTGGATAGTGAGGTTC
ACTB	CATGTACGTTGCTATCCAGGC	CTCCTTAATGTCACGCACGAT

Table S2. Clinical information of 9 relapsed HCC patients and the CD133 expression score in their paired samples

Patient ID	P001	P002	P003	P004	P005	P006	P007	P008	P009
Age	50s	40s	20s	50s	60s	60s	20s	30s	30s
TNM	cT4N0M0 IIIB	pT1bN0M0 IB	cT4N0M1	pT2N0M0 II	pT1bN0M0 IB	—	—	pT1bN0M0 IB	—
Interval between metastatic and primary tumor resection (months)	15	18	27	22	6	23	2	22	29
Treatment performed before primary tumor resection									
Surgery	N	N	N	N	N	N	Y	N	N
Interventional therapy	Y	N	N	N	Y	N	Y	N	N
Targeted therapy	Y	N	N	N	N	N	N	N	N
Immunotherapy	Y	N	N	N	N	N	N	N	N
Chemotherapy	Y	N	N	N	N	N	Y	N	N
Radiotherapy	N	N	N	N	N	N	N	N	N
Treatment performed after primary and before metastatic tumor resection									
Surgery	N	N	N	N	N	N	N	N	N
Interventional therapy	N	N	Y	N	N	Y	N	N	Y
Targeted therapy	N	N	Y	Y	N	N	N	Y	Y
Immunotherapy	N	N	N	N	N	N	N	Y	N
Chemotherapy	N	N	Y	N	N	N	N	N	Y
Radiotherapy	N	N	N	Y	N	N	N	N	Y
CD133 expression score									
Primary tumor	0	0	0	1	0	0	9	0	Unavailable
Paired lung metastasis	0	0	0	2	9	0	9	9	12
Paired intestinal metastasis	—	—	—	—	—	—	—	—	12

Supplementary figures and figure legends

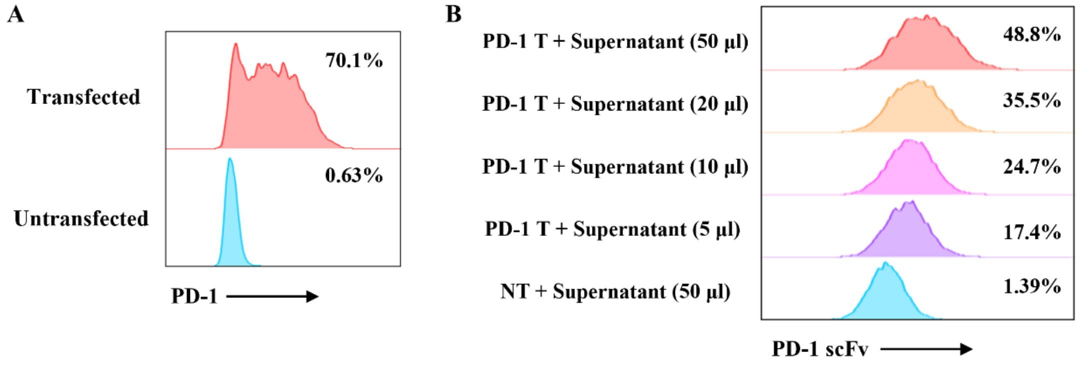

Figure S1. The PD-1 blocking scFv secreted by CAR-T cells can bind the PD-1 antigen on T cells in a dose-dependent manner. (A) PD-1 expression on control and transfected T cells. **(B)** Flow cytometry analysis of the PD-1 blocking scFv binding to NT or PD-1 T cells.

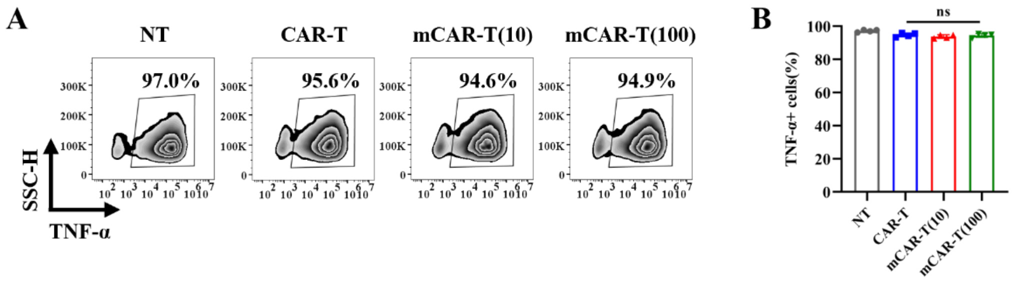

Figure S2. Related to Figure 2. Representative **(A)** and quantitative **(B)** analysis of the percentages of TNF-α^+ T cells in each group. Statistic used two-tailed unpaired *t*-tests (ns: not significant, $p > 0.05$); mean ± SEM are shown (n = 4).

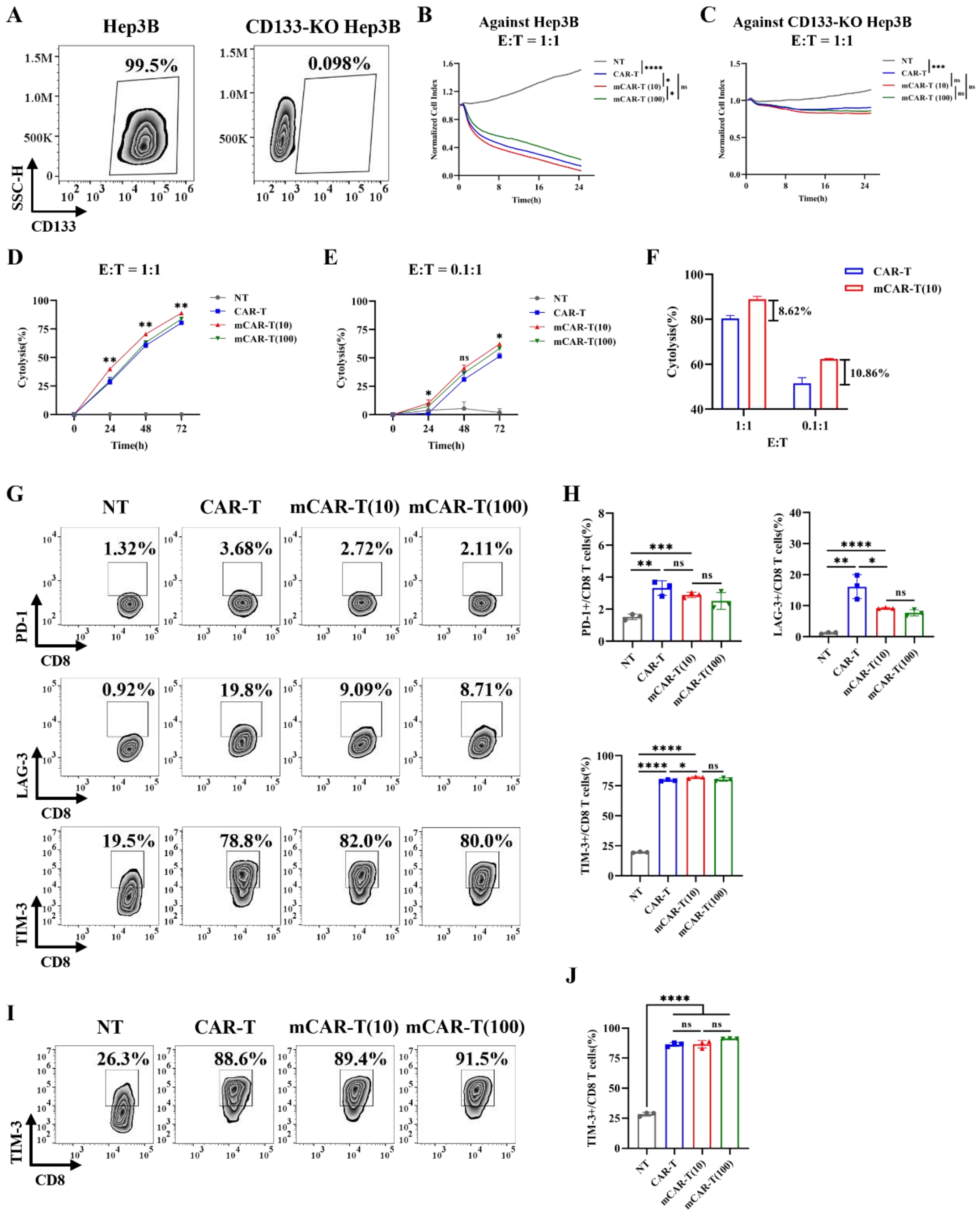

Figure S3. mCAR-T (10) cells exhibit stronger antitumor ability and lower exhaustion marker expression when co-culturing with tumor cells. Related to Figure 3. (A) CD133 expression on wide-type and CD133-KO Hep3B cells. **(B and C)** The cytotoxicity of NT, CAR-T, and mCAR-T cells against Hep3B cells **(B)** and CD133-KO Hep3B cells **(C)** at an E:T ratio of 1:1. Data are presented as the mean of replicates (n = 3), two-tailed unpaired *t*-tests (ns: not significant, $p > 0.05$; *$p < 0.05$; ***$p < 0.001$; ****$p < 0.0001$). **(D and E)** Cytotoxicity analysis of NT, CAR-T, and

mCAR-T cells against CD133-SK-Hep-1 cells at E:T ratios of 1:1 **(D)** and 0.1:1 **(E)**. Statistical analysis was conducted using two-tailed unpaired *t*-tests and showed differences between CAR-T and mCAR-T (10) groups (ns: not significant, $p > 0.05$; *$p < 0.05$; **$p < 0.01$). Mean ± SEM are shown (n = 3). **(F)** Comparison of cytotoxicity between CAR-T and mCAR-T (10) cells after co-culturing with CD133-SK-HEP-1 cells for 72 h at different E:T ratios. Mean ± SEM are shown (n = 3). **(G and H)** Representative **(G)** and quantitative **(H)** analysis showing the percentages of PD-1$^+$, LAG-3$^+$, and TIM-3$^+$ cells gating on CD8$^+$ cells in each group after 48 h of co-culture at an E:T ratio of 1:1. **(I and J)** Representative **(I)** and quantitative **(J)** analysis illustrating the percentages of TIM-3 on CD8$^+$ cells in each group after 72 h of co-culture at an E:T ratio of 1:1. Two-tailed unpaired *t*-tests were used to calculate the *p* value in H and J (ns: not significant, $p > 0.05$; *$p < 0.05$; **$p < 0.01$; ***$p < 0.001$; ****$p < 0.0001$); data are shown as mean ± SEM (n = 3).

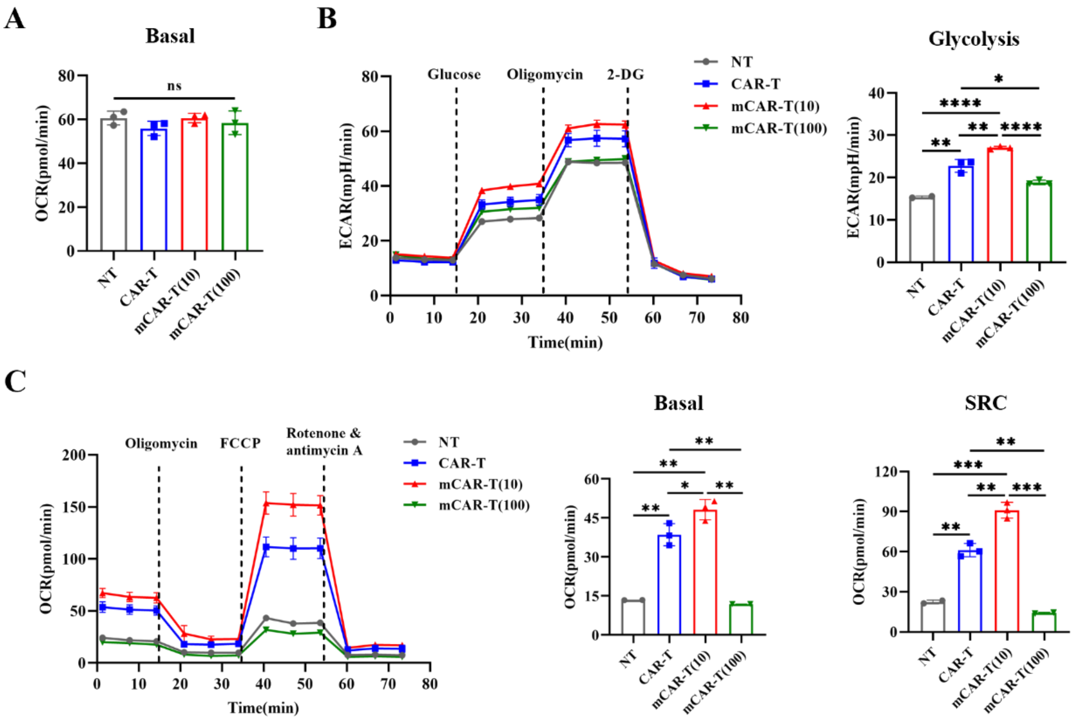

Figure S4. mCAR-T (10) cells exhibit enhanced glycolysis and OXPHOS after 12 h of co-culture with CD133-SK-Hep-1 cells. Related to Figure 5. (A) The basal OCR of each group without antigen stimulation. Statistical analysis was conducted using two-tailed unpaired *t* test (ns: not significant, $p > 0.05$). Mean ± SEM are shown (n =

3). **(B)** The ECAR of each group and the statistical histogram indicating glycolysis measured through Seahorse analysis after antigen stimulation for 12 h. Statistical analysis was performed using two-tailed unpaired *t* test (*$p < 0.05$; **$p < 0.01$; ****$p < 0.0001$). Mean ± SEM are shown (n = 2 or 3). **(C)** The OCR of each group and the statistical histograms of basal OCR and SRC measured through Seahorse analysis after antigen stimulation for 12 h. Two-tailed unpaired *t* test was used to calculate the *p* value (*$p < 0.05$; **$p < 0.01$; ***$p < 0.001$). Data are shown as mean ± SEM (n = 2 or 3).

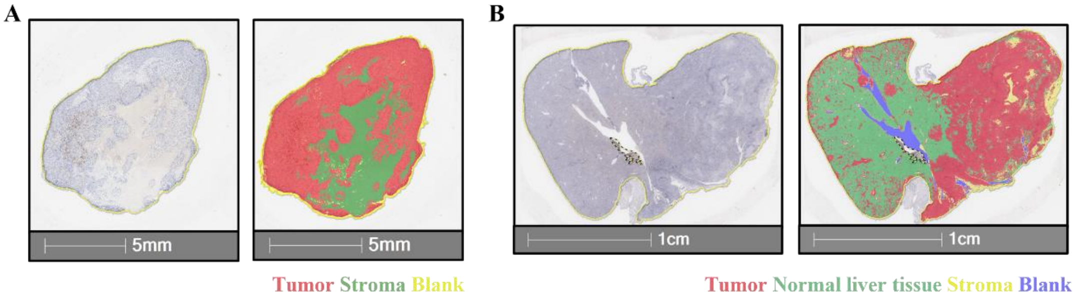

Figure S5. Related to Figure 6 and 7. Representative images illustrating the annotation of IHC slides of subcutaneous xenograft model (**A**) or orthotopic mouse model (**B**) and their digital area classification.
